# How Does Thymine DNA Survive Ultrafast Dimerization Damage?

**DOI:** 10.3390/molecules22010060

**Published:** 2016-12-31

**Authors:** Hongjuan Wang, Xuebo Chen

**Affiliations:** 1 Key Laboratory of Theoretical and Computational Photochemistry of Ministry of Education, Department of Chemistry, Beijing Normal University, Xin-wai-da-jie No. 19, Beijing 100875, China; xuebochen@bnu.edu.cn; 2 Institute of New Energy Materials and Low Carbon Technology, School of Material Science and Engineering, Tianjin University of Technology, Tianjin 300384, China

**Keywords:** thymine DNA, photostability, dimerization, *ab initio* calculation

## Abstract

The photodimerization reaction between the two adjacent thymine bases within a single strand has been the subject of numerous studies due to its potential to induce DNA mutagenesis and possible tumorigenesis in human skin cells. It is well established that the cycloaddition photoreaction takes place on a picosecond time scale along barrierless or low barrier singlet/triplet pathways. However, the observed dimerization quantum yield in different thymine multimer is considerable lower than might be expected. A reasonable explanation is required to understand why thymine in DNA is able to survive ultrafast dimerization damage. In this work, accurate quantum calculations based on the combined CASPT2//CASSCF/AMBER method were conducted to map the excited state relaxation pathways of the thymine monomer in aqueous solution and of the thymine oligomer in DNA. A monomer-like decay pathway, induced by the twisting of the methyl group, is found to provide a bypass channel to ensure the photostability of thymine in single-stranded oligomers. This fast relaxation path is regulated by the conical intersection between the bright S_CT_(^1^ππ*) state with the intra-base charge transfer character and the ground state to remove the excess excitation energy, thereby achieving the ground-state recovery with high efficiency.

## 1. Introduction

Ultraviolet radiation ranging from 200 to 300 nm in wavelength can induce DNA mutagenesis and possible tumorigenesis in human skin cells due to the dimerization reaction between the two adjacent thymine bases within the same strand [1]. The formation of the cyclobutane pyrimidine dimer (CPD) has a close relationship with human health as it can influence gene replication, transcription and expression and cause apoptosis, immune suppression and carcinogenesis [2,3,4]. There has been intense research interest in various relevant aspects of the photophysical and photochemical properties of thymine monomers [5,6,7,8,9,10,11,12,13,14,15,16,17], oligomers [11,13,17,18,19,20,21,22] and DNAs [7,21,22,23,24,25,26,27,28] for a wide range of systems in past decades. Experimentally, the photoexcited thymine monomer was observed to show an ultrafast decay with a time constant of less than 1 ps [5,8,9,10,17,29]. The electronic structure and dynamic calculations suggest that it relaxes to the ground state first along a barrierless route in the bright excited state and then through the conical intersection (CI) between ^1^ππ* and ground states [12,15,16,30]. This sub-picosecond internal conversion has been considered as the hallmark of monomer excited state for the photophysics of DNA or its constituent bases, which is also observed in single-stranded (dT)_n_ oligomers [13,29]. In contrast, the double-stranded (dA)_n_·(dT)_n_ or single-stranded (dA)_n_ oligonucleotides exhibit an excited-state lifetime longer than 100 ps [29,31,32,33]. The slow deactivation of photo-excited adenine DNA has been theoretically rationalized in our group by multi-configurational perturbation theory [34], in which the direction of charge transfer for the bright excitation state is found to be completely inverted due to dipole interaction between six and five member rings in stacked adenine bases. This significant alternation for the nature of the initial population state leads to a considerable increase in the barrier for excited state decay through the structural deformation of C–H or NH_2_ group twisting, which accounts for why the excited-state deactivation process of the adenine oligomers is significantly different from that of the monomer.

For the photochemistry of thymine multimers, there has also been longstanding controversy regarding the singlet vs. triplet nature of the excited state as the origin of T<>T CPD formation. A singlet dimerization mechanism was proposed to proceed along a ^1^ππ* excited state [18,19,20], in which the CPD photoproduct was observed to be fully formed within ~1 ps by using time-resolved spectroscopic techniques. The corresponding theoretical calculations demonstrated that ultrafast [2 + 2] cycloaddition photoreaction is regulated by the non-adiabatic relay of a CI between the ^1^ππ* excited and ground states [35,36,37]. Conversely, the combined femtosecond broadband time-resolved fluorescence and transient absorption spectroscopy investigations suggest that the triplet T_1_ state could be a very efficient precursor to yield CPD in a thymine oligomer, since the intrastrand T_1_ self-quenching reaction is greatly facilitated in (dT)_20_ (featured by ~140 ps time constant) compared with long lived triplet state of thymine monomer (up to ~4 ns) [13]. Consistently, a favorable energy barrier was found for the CPD generation in triplet state pathways at density functional [38] and multiconfigurational perturbation [39] levels of theory, respectively, using a thymine dimer model. Although the cycloaddition photoreaction was determined to take place on a picosecond time scale (1–140 ps) along barrierless or low barrier singlet/triplet reaction pathways from experimental and theoretical perspectives, the observed dimerization quantum yield in different thymine multimers is considerable lower than expected. For example, the quantum yield of CPD formation is 2%–3% in isolated DNA (with respect to thymine) and in single-strand, all-thymine oligodeoxynucleotides [17,19,40,41,42,43,44] and 1%–2% for TpT [17,19,41,42] while the highest one (10%) is observed for T_L_pT_L_ [19,45]. These detected data indicate that most thymine bases can survive dimerization damage, which apparently conflicts with the notion of barrierless or low barrier pathways for cycloaddition photoreaction. Therefore, a reasonable explanation is required to address this contradiction on the basis of quantitatively theoretical or experimental evidences, although this is preliminarily speculated to be due to the rareness of reactive conformations in a thermal ensemble [18,19,22,40].

## 2. Results and Discussion

### 2.1. Ultrafast Decay of Thymine Monomer in Bright S_CT_(^1^ππ*) and T_CT_(^3^ππ*) States

**Figure 1:**
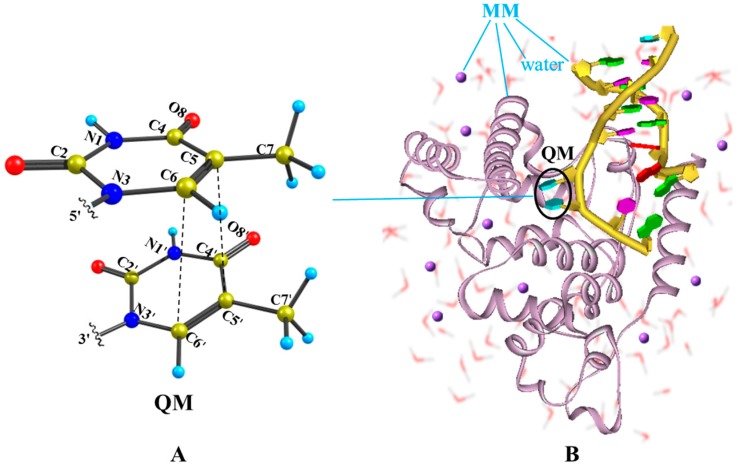
Illustration of the QM/MM computational protocol adopted in this work for finding the MEPs of thymine oligomer in DNA and solvent environment. (**A**) The QM subsystem includes two adjacent thymine bases; (**B**) The remaining of DNA bases, amino acid residues, crystal water molecules and counter-ion Na^+^ are treated by the molecular mechanics.

The electronic transition of thymine monomer in aqueous solution to the bright state is of ^1^ππ* character with a significant charge transfer (CT) character and the relatively large oscillator strength (*f* = 0.30). According to the electronic population analyses (see Table S2 in Supplementary Materials), this excitation originates from electron promotion from the C5=C6 double bond to the C4=O8 carbonyl group (See Figure 1 for number scheme). The charge translocation calculations reveal that 0.2 atomic charge migrates between two moieties by using the Mulliken population and an appropriate fragment strategy (see Supplementary Materials). The vertical excitation energy calculated here is 4.88 eV for thymine monomer in polar solvent, which is 0.01~0.56 eV red shift compared with that in gas phase values in previous computations [15,16,35]. This confirms the CT nature for the bright state excitation that therefore is denoted as S_CT_(^1^ππ*) hereafter. The computational excitation energy for the bright S_CT_(^1^ππ*) is consistent with the experimental wavelength of 266 nm (4.67 eV) in the buffered aqueous solution [13], which indicates that the S_0_→S_CT_(^1^ππ*) transition is responsible for the initial population under the experimental condition. Unlike the case of the S_CT_(^1^ππ*) state, the FC excitation of the S_NP_(^1^nπ*) state in solution is 0.08 eV blue shifted compared with that in gas phase due to the formation of diradical configuration [16]. As a result, the ^1^ππ*/^1^nπ* energy gap gets narrowed in solution with respect to the case in gas phase. 

**Figure 2:**
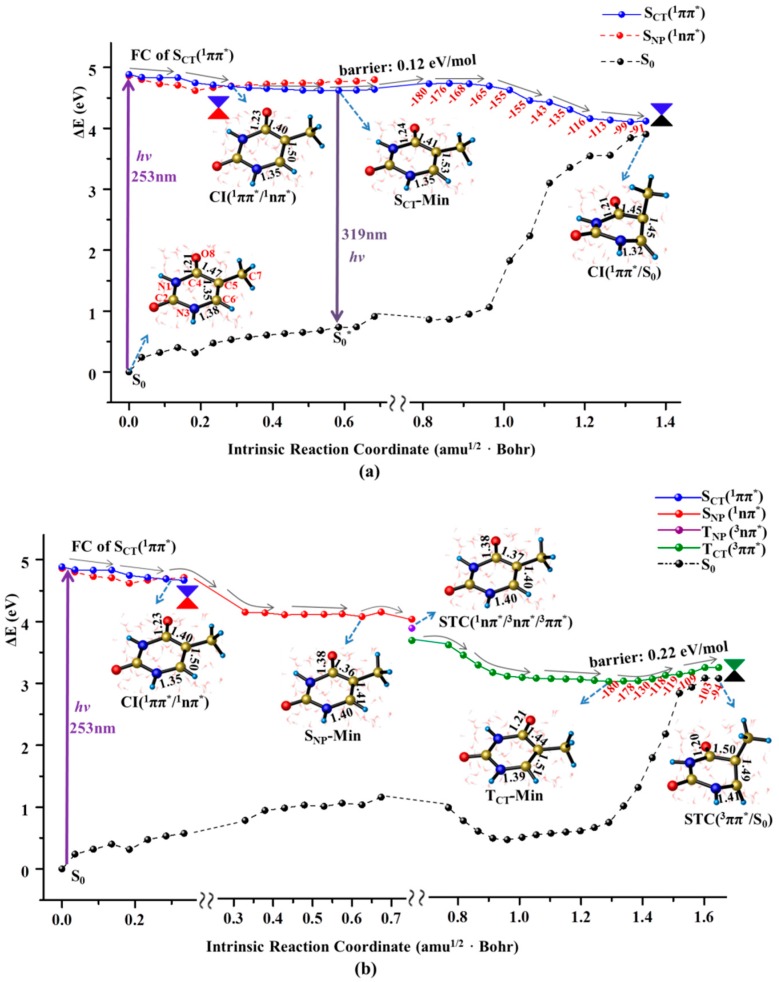
MEPs of two competitive deactivation paths for the thymine monomer in aqueous solution photo-initiated by 253 nm light in the bright S_CT_(^1^ππ*) state (**a**) and T_CT_(^3^ππ*) state (**b**). Schematic structures are shown with the selected bond lengths (in Å) and the changes of N1C4C5C7 dihedral angle are given along the relaxation path (the red numbers in degree).

As illustrated in Figure 2a, the initial relaxation of the photo-excited thymine monomer in aqueous solvent is characterized by the structural changes of elongated C5=C6 and C4=O8 bonds, which reflects CT nature along the desired direction for the FC excitation. These structural adjustments let S_CT_(^1^ππ*) state to exactly intersect with a n→π* excited state, S_NP_(^1^nπ*), that originates from the one electron promotion from lone pair of O8 to the π* orbital of conjugated ring. This conical intersection is referred as CI(^1^ππ*/^1^nπ*) and is energetically locates at 0.2 eV below FC of S_CT_(^1^ππ*) state, which exhibits the structural feature of weakened C5=C6 (1.50 Å ) and C4=O8 (1.23 Å) bonds compared with those of normal double bond character (1.35/1.21 Å) in the FC of S_CT_(^1^ππ*) state. An important consequence for the existence of conical intersection is to provide a bypass channel to complete non-radiative decay through S_NP_(^1^nπ*) state intermediate funnelled by the non-adiabatic relay of CI(^1^ππ*/^1^nπ*) (*vide infra*). A flat energy profile takes aqueous thymine monomer decay to its minimum of S_CT_(^1^ππ*) state, S_CT_-Min, in which C5=C6 and C4=O8 bonds undergoes the further elongation with the concomitant slight reinforcement of C4–C5 and N3–C6 bonds. A tiny barrier (0.12 eV/mol) is encountered to have a little possibility to trap the system in S_CT_-Min, which indicates an unfavorable radiative deactivation for the excited state decay of thymine monomer in aqueous solution. Consistently, an extremely low quantum yield (9 × 10^−5^) of fluorescence emission at 329 nm was observed in experimental measurement [13]. The vertical emission energy of S_CT_-Min→S_0_* is computed to be 3.88 eV (319 nm), which is in good agreement with the experimental value (329 nm) [13].

The eliminated constraint resulting from the elongation of C5=C6 bond triggers the motion of methyl group twisting along downhill relaxation path in S_CT_(^1^ππ*) state. As shown in the right panel of Figure 2a, this structural deformation causes a noticeable energy increase of the ground state. Meanwhile, the energy profile of S_CT_(^1^ππ*) state goes down and eventually reaches the conical intersection between these two states, referred as CI(^1^ππ*/S_0_), at which the N1–C4–C5–C7 dihedral angle is −91.0°. The CI(^1^ππ*/S_0_) from our calculation is energetically 4.11 eV above the ground state of the thymine monomer, which agrees with the previous results of 4.0 eV in gas phase [16,46].

The aforementioned CI(^1^ππ*/^1^nπ*) bifurcates the excited state relaxation to funnel the thymine monomer into the surface of dark S_NP_(^1^nπ*) state starting from the bright S_CT_(^1^ππ*) state (see Figure 2b). The initial decay in the S_NP_(^1^nπ*) state is characterized by the elongation of carbonyl C4=O8 group as indicated by the bond length of 1.23 Å at CI(^1^ππ*/^1^nπ*) to 1.38 Å at the minimum of S_NP_(^1^nπ*) state, S_NP_-Min. With these structural deformation, the thymine monomer decays to the three surface crossing of STC(^1^nπ*/^3^nπ*/^3^ππ*) whose structure is very close to that of the S_NP_-Min. Like observations in many aromatic carbonyl compounds [47,48], the existence of three surface crossing allows an effective intersystem crossing (ISC) from ^1^nπ* to T_CT_(^3^ππ*) as an El-Sayed-type manner. It should be noted that the direct population of the ^1^nπ* state for the solvated thymine is quite small and has been challenged to not occur at all [49,50]. Moreover, we fail to find the conical intersection between S_NP_(^1^nπ^*^) and ground states, which rules out the possibility of the direct deactivation from the S_NP_(^1^nπ^*^) state. Our computational results show that the nπ* state is found to play a mediating role in bifurcating the excited state relaxation into the triplet state through ISC conversion. Once thymine monomer is populated in the T_CT_(^3^ππ*) state that is verified to have a diradical configuration distributed around the C5 and C6 atoms, the decay is characterized by the structural recovery of an elongated C4=O8 carbonyl bond caused by the relaxation in S_NP_(^1^nπ*) state. Meanwhile, C4–C5 and C5–C6 bonds undergo further elongation and thus relax to the minimum of T_CT_(^3^ππ*) state, T_CT_(^3^ππ*)-Min, removing the structural constraint for the subsequent methyl twisting. Like that in S_CT_(^1^ππ*) state, the methyl twisting proceeds smoothly along a flat energy profile ranging from 2.98 to 3.20 eV with respect to the zero level of ground state minimum. As a result, thymine monomer relaxes to the singlet-triplet crossing of T_CT_/S_0_ completing the fast ground state recovery. Thus, the dark S_NP_(^1^nπ*) state servers as an effective bifurcation to relay the singlet and triplet decay channels induced by the methyl twisting, ensuring the photo-stability of thymine monomer.

As illustrated in Figure 2b, the structural features and energetic levels between the S_NP_(^1^nπ*)-Min and the subsequent ^1^nπ*/^3^ππ*/^3^nπ* crossing are indistinguishable, suggesting a mixture character of the ^1^nπ* intermediate state. The present computations provide solid evidence that the found ^1^nπ* intermediate state has a singlet origin mixed with a significant ππ* triplet character. Consistently, an intermediate termed by “doorway state” with the similar mixture nature was experimentally proposed to rely the singlet and triplet relaxation pathways [13]. The calculated SOC for ^1^nπ*→^3^ππ* conversion in aqueous solution (35.6 cm^−1^) is one fold smaller than that in gas phase (61.0 cm^−1^) [16], which suggests a more effective decay channel along singlet path of S_CT_(^1^ππ*) state with CT character that can be stabilized by polar environment.

### 2.2. Nonradiative Decay and Dimerization of Thymine Oligomer in Bright S_CT_(^1^ππ*) and T_CT_(^3^ππ*) States

**Figure 3:**
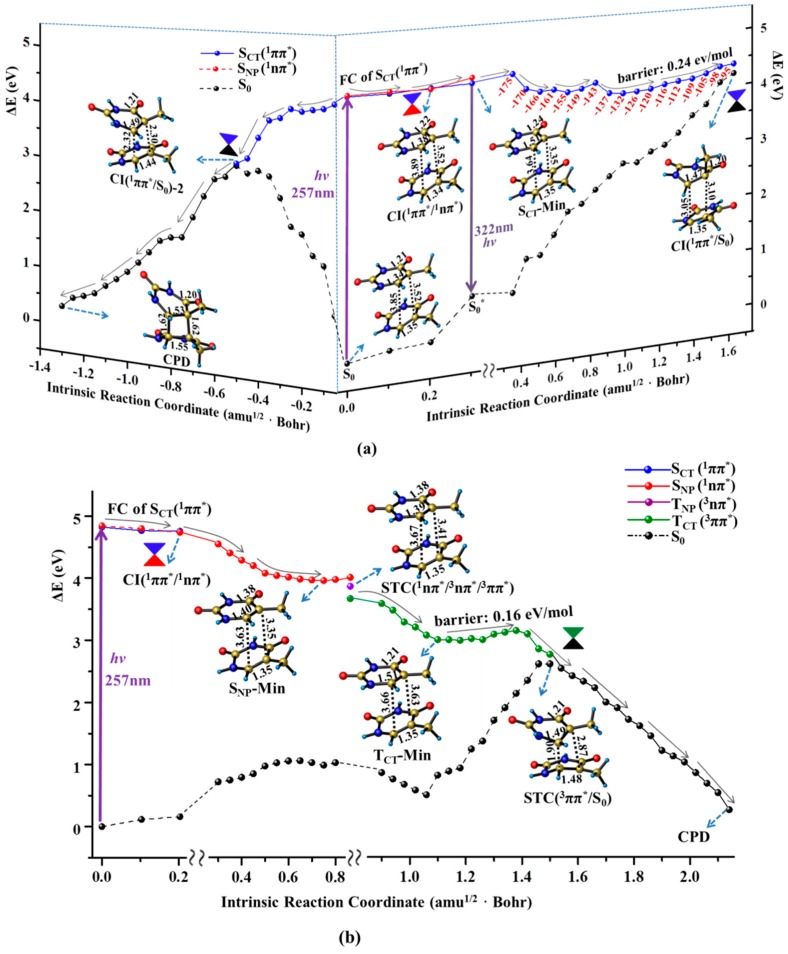
MEPs of three competitive deactivation paths for the thymine oligomer in DNA photo-initiated by 257 nm light in the bright S_CT_(^1^ππ*) (**a**) and T_CT_(^3^ππ*) state (**b**), obtained at the CASPT2//IRC//CASSCF(14e/11o)/Amber QM/MM level of theory. Schematic structures are shown with the selected bond lengths (in Å) and the changes of N1C4C5C7 dihedral angle are given along the singlet sate relaxation path (the red numbers in degree).

Like the case of thymine monomer, the bright state excitation of thymine oligomer is also attributed to the single base CT transition originating from C5=C6 double bond to the C4=O8 carbonyl group (see Supplementary Materials). Moreover, in accordance with the experimental measurements [13], the calculated maximum absorption of S_0_→S_CT_(^1^ππ*) with peak at 257 nm is very close to that of thymine monomer (253 nm). The similar Frank-Condon (FC) population causes the decay of thymine oligomer in S_CT_(^1^ππ*) state to procced as the monomer like manner. As shown in the right panel of Figure 3a, the structural deformation of methyl twisting is immediately triggered along a flat pathway. The large inter-base distance (3.5–4.0 Å) provides the spatial allowance to boost the methyl distortion, thus removing the excess excitation energy. The thymine oligomer ultimately evolves in the energetic degeneracy region between S_CT_(^1^ππ*) and ground states, i.e., CI(^1^ππ*/S_0_), that functions as a non-adiabatic relay resulting in an effective access of the ground state when the photo-activated single base undergoes a significant ring puckering motion (ca. 80.0° methyl twisting), by overcoming a small barrier (0.24 eV). As a result, a fast ground sate recovery of the photo-excited thymine oligomer can be achieved through the monomer like decay associated with the spatially allowed methyl twisting. Here, the computed barrier for the ground state recovery of the thymine oligomer is two-fold larger than that of the thymine monomer. This may arise from the steric hindrance of the MM environment. And a related analysis of the QM and MM parts has been conducted in Conti’s paper that reveals a long-lived excited state [51]. Moreover, the energy barrier is computed from the planar minimum obtained at the CASSCF level which is different from the results computed at the TD-DFT and CASPT2 level. This is mainly due to the lack of the dynamic correlation for the CASSCF, which has been discussed in the previous study [52,53,54].

Besides the monomer like decay, the neighboring thymine unit likely gets involved in the relaxation of thymine oligomer along the singlet excited state pathway. As shown in the left panel of Figure 3a, the C5=C6 double bond is gradually elongated to the single bond (1.34→1.51 Å) exhibiting an obvious diradical character along the initial FC→S_CT_-Min decay, which immediately triggers the attack reaction of C5–C6 bond towards the C5′=C6′ double bond of the neighboring thymine. The dimerization proceeds in a concerted manner in which the distances of C5–C5′ and C6–C6′ are decreased simultaneously leading to the distort deformation in two neighboring bases. As a result, the energy level of the ground state undergoes a drastic increase and eventually intersects with the S_CT_(^1^ππ*) state, denoted as CI(S_CT_/S_0_)-2 hereafter. Passing through the non-adiabatic relay, the C5–C5′ and C6–C6′ bonds are further shortened in the ground state, thereby generating the final CPD product. Thus, the ultrafast dimerization of thymine bases can be achieved along the downhill path of the singlet state, which is consistent with the experimentally measured time scale (<1 ps) [18]. The drastic structural deformation involving the two neighboring bases is required for the CPD production in the singlet state which cannot compete with the ultrafast ground state recovery via the monomer-like decay of methyl twisting. Consistently, previous studies have observed a bi-exponential sub-ps decay experimentally and shown that the arrangements between the stacked nucleobases play an important role on the preferred decay path [49,53,55]. Meanwhile, the ring-puckering motion in similar biology system has been reported before [56]. Recently, a nonadiabatic molecular dynamics study rationalized the low CPD formation yield based on the topology of the CI connecting the S_1_/S_0_ in the dimerization [57].

Like the case of the thymine monomer in the triplet state, three surface crossings of STC (^1^nπ*/^3^nπ*/^3^ππ*) are repeatedly determined in the early relaxation of thymine oligomer which allows the ^1^nπ* state to act as the doorway of the intermediate relay, thus resulting in access to the T_CT_(^3^ππ*) state. Once the thymine oligomer is populated in the T_CT_(^3^ππ*) state, the dimerization between the two adjacent thymine bases is immediately triggered to proceed in a stepwise manner. The C6–C6′ bond is initially formed in the triplet state by overcoming a tiny barrier (0.16 eV/mol). With the first C–C bond formation, the energy level of the ground state increases rapidly which approaches the T_CT_(^3^ππ*) state, leading to the singlet–triplet crossing of STC(T_CT_/S_0_). The spin density analysis presents a diradical character at the STC(T_CT_/S_0_) with two unpaired electrons at the C5 and C5′ atoms, which can facilitate the occurence of spin inversion with high efficiency, giving rise to an excellent precursor for the subsequent dimerization reaction. Finally, the C5–C5′ bond is generated by an ultrafast combination of the diradical in the ground state, leading to the product of CPD. Therefore, the spin-forbidden ^1^nπ*→^3^ππ* ISC becomes the rate-determining step for the dimerization reaction in the triplet manifold which unlikely compete with the photochemical and photophysical decay in the singlet state. Consistently, a minor amount of the CPD photoproduct was observed in the time-resolved spectroscopy [13].

## 3. Computational Methods

In this work, a combined quantum mechanical/molecular mechanical (QM/MM) approach, at the CASPT2//CASSCF/AMBER level of theory, is used to compute the minimum energy profiles (MEPs) along unbiased reaction coordinates in possible electronic states to model the relaxation paths of the thymine oligomer in DNA and solvent environment. For comparison, the corresponding decay channels of an isolated thymine in aqueous solution are also examined at the same level of theory. The computational protocol adopted in this work has been validated to be a powerful method for investigating complicated mechanisms regarding the slow deactivation in adenine DNA [34] and the photorepair of cyclobutane pyrimidine dimer (CPD) splitting in thymine oligomer [58].

In recent QM/MM studies, the normal single- and double-strand DNA were chosen as the initial computational models [57,59]. Here, a repaired double-strand DNA with a bent thymine dimer was used, facilitating the CPD formation. The modelling system was initially taken from the RCSB protein data bank (PDB) under code name 1TEZ chain A [60]. To reduce the computational cost, residues 1 to 237, which are far from the reaction center, were removed from the N-terminal, resulting in a starting structure with 5402 atoms. To keep the whole system electrically neutral, 13 Na^+^ counter-ions were added. Figure 1 shows the QM/MM computational protocol, in which the QM subsystem includes two adjacent thymine bases, while the rest of the DNA bases, amino acid residues, crystal water molecules, and counter-ions are treated with the MM approach. The ab initio calculations here were primarily performed at the CASSCF level of theory with a total of 14 electrons in 11 active orbitals for the stacked thymine bases in DNA and 14 electrons in 10 active orbitals for the thymine monomer in aqueous solution, referred to as CASSCF(14e/11o) and CASSCF(14e/10o), respectively, with a cc-PVDZ basis set. In previous studies, Roca-Sanjuán et al. have reported the photo-deactivation processes for the thymine dimer and revealed that the initial excitation has to be localized on a single nucleobase. The computations were conducted at the CASSCF level of theory with the balanced (12, 12) active space, comprising six orbitals for six electrons for each of the moieties present in the dimer [61,62]. Here, in order to reduce the computational cost for the thymine dimer, the active orbitals are mainly concentrated on a single part. All critical points of minima, conical intersections and singlet/triplet state crossings were rigorously obtained by full system single root or multi-root state-averaged optimizations. The MEPs were mapped by intrinsic reaction coordinate (IRC) computations to connect above critical points in different excited and ground states. For the damaged path in the bright S_CT_(^1^ππ*) state, the modified starting geometry with shorter interbase distance was used, while the Franck-Condon structure was applied to calculate the photophysical path. To consider the dynamic electron correlation effects, the single-point energy of optimized geometries in the above computations was recalculated at the multiconfigurational second-order perturbation theory level (CASPT2) using a six-root state-averaged zeroth-order wave function [63,64,65,66,67]. As discussed in previous works [61,68] the energy gaps for CIs at the CASPT2 level are slighter larger than those at the CASSCF level, but are still within an acceptable range (<0.2 eV). The calculations were performed using the GAUSSIAN [69] and MOLCAS [70] program packages. For the MM part, the AMBER force field ff99 [71] was employed using the TINKER [72] tool package. A hydrogen link-atom scheme was used to saturate the valence of the QM subsystem, where the bonds between the QM and MM region were cleaved (indicated by the wavy lines in Scheme S1). To reduce the strong interaction between a link atom and the closest MM point charge, the latter was set to zero [73]. As a compensation, some point charges of the MM atoms were re-parameterized as summarized in Table S1 of the Supplementary Materials. For more computational details see Section 1 of the Supplementary Materials.

## 4. Conclusions

In summary, extensive theoretical investigations have been performed at CASPT2//CASSCF/AMBER level of theory to provide the mechanistic understanding of photostability for the thymine monomer in aqueous solution and the thymine oligomer in DNA. Our computational results show explicitly that the doorway nπ* state is found to play a decisive major role in bifurcating the excited state relaxation into the predominant singlet pathway and a minor pathway through ISC conversion to the ^3^ππ* state to achieve the ground state recovery of thymine monomer. A monomer-like decay pathway in the singlet state is demonstrated to provide a bypass channel to ensure the photostability of thymine in single-stranded oligomers through the structural deformation of methyl twisting. The present computational results contribute the underlying molecular mechanisms to understand the photostability of thymine in the DNA environment.

## Supplementary Materials

Supplementary materials can be accessed at: http://www.mdpi.com/1420-3049/22/1/60/s1.
