# Supplementary material for: How Does Thymine DNA Survive Ultrafast Dimerization Damage?
Source: Molecules. 2016 Dec 31;22(1):60. doi: 10.3390/molecules22010060 (PMC6155609; doi:10.3390/molecules22010060)
Supplement: Supplementary file 1 [file molecules-22-00060-s001.pdf]

Supplementary Materials: How Does Thymine DNA Survive Ultrafast Dimerization Damage?

Hongjuan Wang and Xuebo Chen

1. Computational Details

1.1. Model Setup

In this work, two computational models were built. The first one is comprised of a thymine monomer surrounded by water molecules. And the second one is taken from the RCSB Protein Data Bank (PDB) with code name 1TEZ chain A [1]. that containing repaired double-helical DNA, amino acid residues, water molecules and $FADH^-$. To reduce the computational burden, residues 1 to 237, which are far from the reaction center, were removed from N-terminal. Thirteen Na^+ counterions were added using the tleap module of the AMBER10 package [2] to neutralize the system in accordance with experimental conditions. The 285 crystal water molecules in the protein were kept and the AMBER-parm99 force field [3] was employed for the whole system.

1.2. Equilibrium Molecular Dynamics (MD)

The initially constructed systems were equilibrated for 1 ns using classical canonical MD simulations (at 298 K, NVT ensemble). A cutoff radius of 9 Å was used for the real space electrostatic interactions and the van der Waals terms. All MD simulations were performed with the TINKER4.2 package [4]. A cluster analysis of the sampled snapshots generates the appropriate starting structure for the QM/MM calculation.

1.3. QM/MM Computational Protocol

Scheme S1 shows the chosen QM/MM partitioning. To explicitly describe the deactivation paths for the thymine monomer in water box, a small QM1 part containing one thymine molecule was used. To comprehensively account for the decay and dimerization processes for the thymine oligomer, the two adjacent thymine bases were selected as the QM2 part, while the rest of the DNA bases, amino acid residues, crystal water molecules, and counterions were treated with the MM approach. The boundary separating the QM and MM regions was treated by the hydrogen link-atom scheme (see the wavy lines in Scheme S1). To reduce the strong electrostatic interactions between a link atom and its nearest MM atoms, the weight-consistent reparameterization scheme introduced by Olivucci et al. was adopted to adjust the MM point charges near the QM/MM boundaries [5–8]. Specifically, the nearest point charge was set to zero and the other neighboring MM point charges were re-parameterized (see Table S1). For the remaining MM atoms, standard force-field point charges were used.

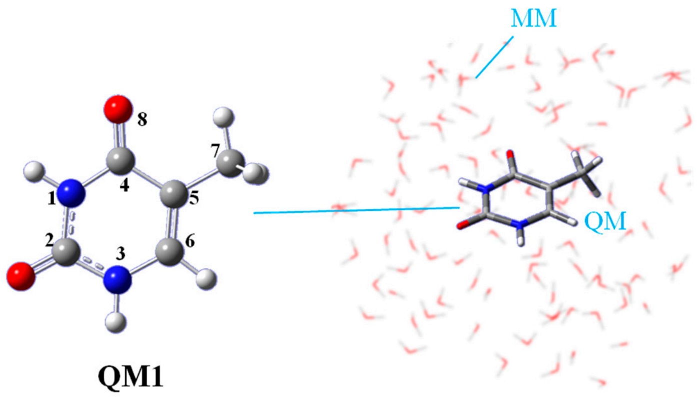

(**a**)

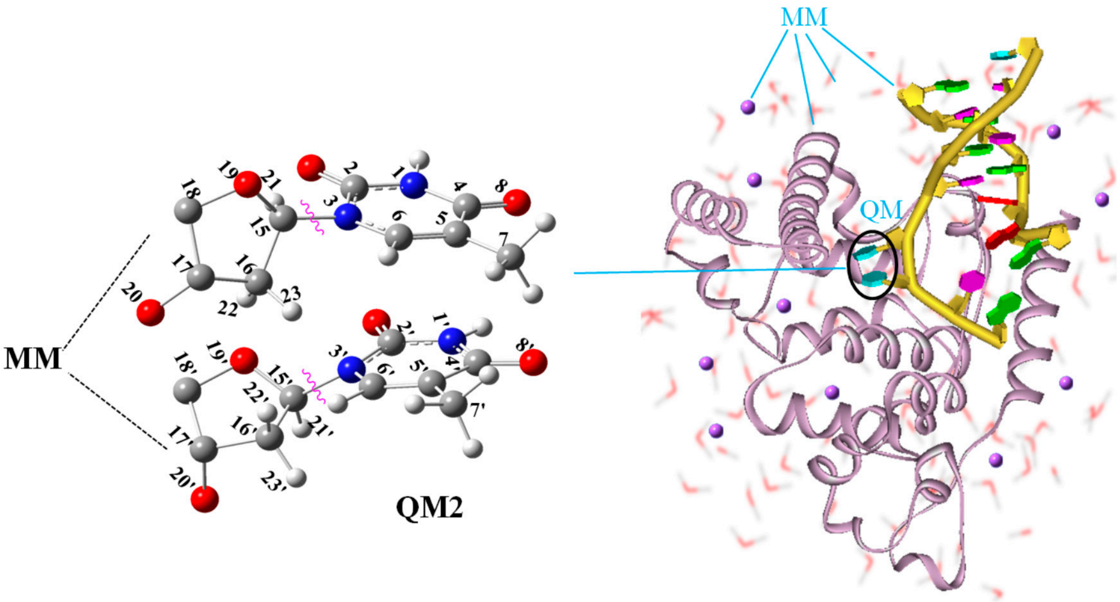

(**b**)

Scheme S1. The chosen QM/MM partitioning: (**a**) the QM1 subsystem includes the thymine monomer; (**b**) the QM2 subsystem includes two adjacent thymine bases, while the MM subsystem includes the rest of the DNA bases, amino acid residues, crystal water molecules, and counterions. See the text for details.

Table S1. Re-parameterized point charges (a.u.) for the MM atoms near the QM/MM boundary.

**C15**	**0.0000**	**C15′**	**0.0000**
C16	−0.0754	C16′	−0.0754
C17	0.0753	C17′	0.0753
C18	0.1649	C18′	0.1649
O19	−0.3431	O19′	−0.3431
O20	−0.5192	O20′	−0.5192
H21	0.1824	H21′	0.1824
H22	0.0818	H22′	0.0818
H23	0.0818	H23′	0.0818

1.3.1. QM Method

The calculations of the QM parts were conducted at the complete active space self-consistent field (CASSCF) level of theory [9,10]. with the cc-PVDZ basis set. For the thymine monomer, the ab initio calculations were primarily performed at the CASSCF level of theory with a total of 14 electrons in 10 orbitals (14e/10o). The active orbitals include the O4 lone-pair orbital, all π orbitals and their corresponding π* orbitals (see Figure S2). For the thymine oligomer, 14 electrons in 11 orbitals were chosen as the active space, which includes C5–C6 (C5′–C6′) π/π* orbitals, C4–O8 (C4′–O8′) π/π* orbitals, O8 lone-pair n orbital and the delocalized π orbitals on the 5′-thymine. All of these orbitals in the active space are shown schematically in Figures S3. Geometry optimizations were performed using a 2-root state-averaged CASSCF approach (S_0 and S_{CT}, equal weights) for the S_{CT} state and a state-specific approach for the S_0 and T_1 state. To consider dynamic electron correlation effects, the single-point energy of the optimized geometries in the above computations was recalculated at the multi-configuration second-order perturbation (CASPT2) level of theory [11,12] based on the zeroth-order six roots state-averaged CASSCF wave functions. These calculations were performed without an ionization potential-electron affinity (IPEA) shift but included an imaginary energy-level shift of 0.2 a.u. to avoid intruder state problems.

1.3.2. Vertical Excitation Energies

Vertical excitation energies, oscillator strengths and transition dipole moments to the lowest five excited singlet states of the QM part at the Franck-Condon (FC) point were computed using the CASPT2//CASSCF and CASSI//CASSCF methods at the CASSCF-optimized S_0 minimum.

1.3.3. Optimizations of Minima, Conical Intersections and Paths

Local minima on the excited and ground states were obtained by CASSCF optimizations. The location of conical intersections and singlet/triplet crossings was assessed on the basis of the computed energy gaps for the optimized structures. At the same computational levels, the minimum energy profiles (MEPs) were mapped by intrinsic reaction coordinate (IRC) computations [13,14] to connect above critical points in several possible excited and ground states. The single point energy calculations were carried out at the CASPT2 level of theory, based on optimized geometries using the CASSCF method. Therefore, the MEPs were eventually computed at the CASPT2//IRC/CASSCF level of theory along the unbiased reaction coordinates to gain insight into how the deactivation for the thymine monomer and thymine oligomer takes place.

1.3.4. Packages

The CASSCF calculations were performed using GAUSSIAN03 [15]. The CASPT2 and CASSI calculations were performed using MOLCAS7.6 [16], whereas the MM calculations were conducted under the AMBER99 [3] force field using TINKER4.2 package [4]. The interface between the QM and MM parts was coded by Ferré et al. and included in the Molcas program [17].

2. Charge Translocation Calculations

To further explore the properties of thymine monomer and thymine oligomer in the excited state, a charge translocation calculation was performed based on Mulliken charge population and an appropriate fragment partitioning strategy. As shown in Figure S1, for the thymine monomer, the link nitrogen group and its adjacent –CH group are defined as part I, while the rest part in the ring are defined as part II. For the thymine oligomer, the unexcited thymine base are included as part II. The charge distributions were obtained using a full Mulliken population analysis at the CASPT2//CASSCF level of theory. Table S1 presents the Mulliken charge distributions of part I and II in the ground (S_0) and $S_{CT}(^1\pi\pi^*)$ state upon the photo-excitation of thymine monomer and thymine oligomer.

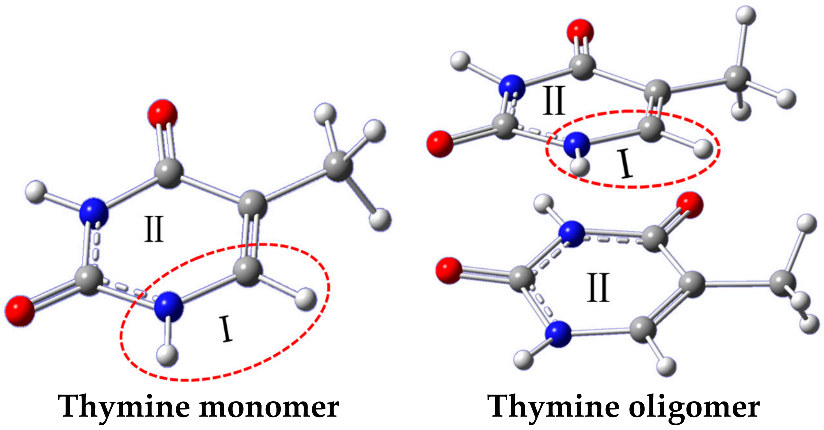

Figure S1. The scheme of fragment partition for charge translocation is shown, for the thymine monomer, the link nitrogen group and its adjacent –CH group are defined as part I, while the rest part in the ring are defined as part II. For the thymine oligomer, the unexcited thymine base are included as part II.

Table S2. Mulliken charge distribution of thymine monomer and thymine oligomer in part I and II in the ground (S_0) and the $S_{CT}(^1\pi\pi^*)$ state upon the photo-excitation. (unit: e).

		S_0	$S_{CT}(^1\pi\pi^*)$	Charge Translocation
Thymine monomer	Part I	0.1274	0.3193	0.1919
	Part II	−0.1274	−0.3193	
Thymine oligomer	Part I	0.0830	0.2616	0.1786
	Part II	−0.0830	−0.2616	

3. Selected Orbitals in the Active Space

Diagram of selected orbitals in the active space for the thymine monomer and thymine oligomer.

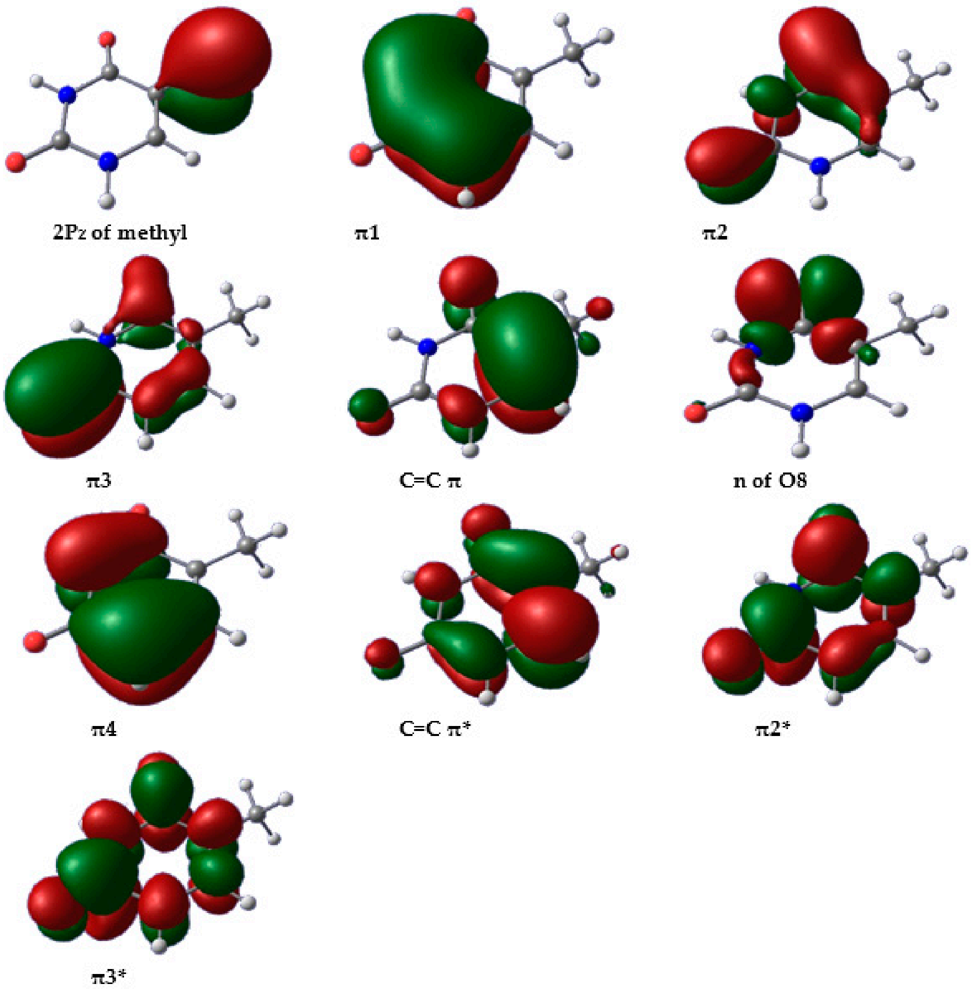

Figure S2. Molecular orbitals of thymine monomer used in defining the active space for the CASPT2//CASSCF (14e/10o) calculations.

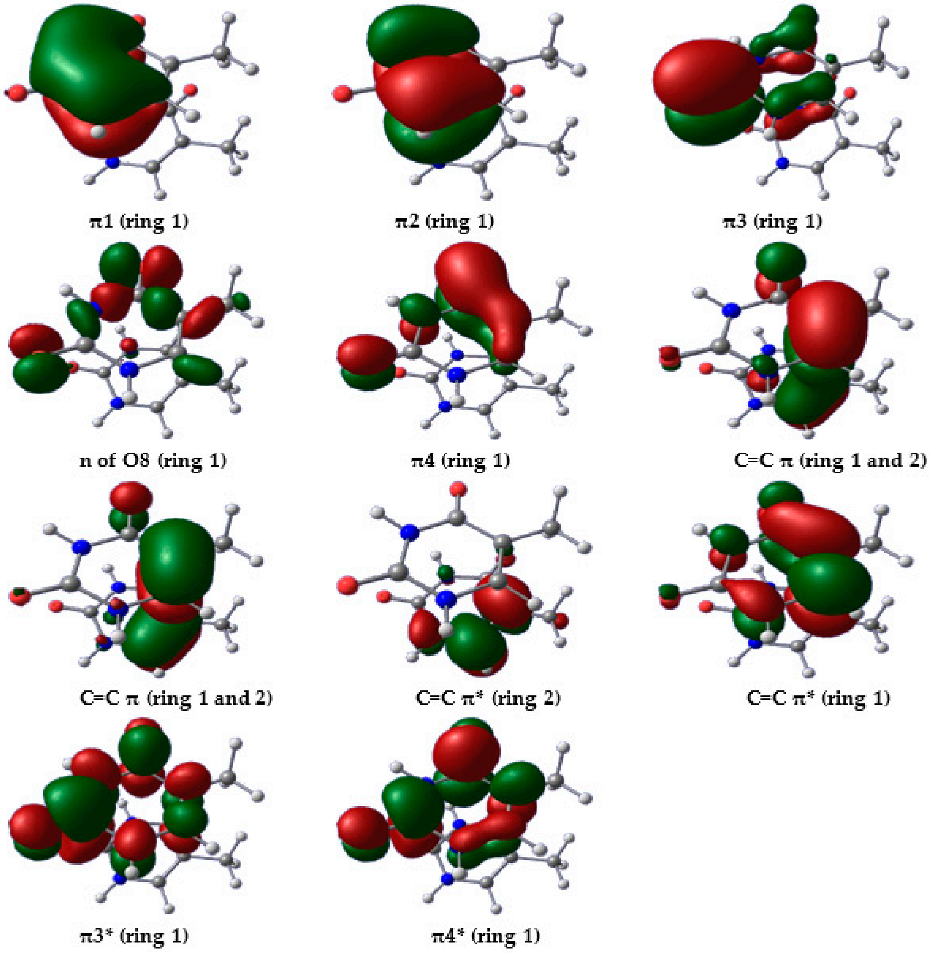

Figure S3. Molecular orbitals of thymine oligomer used in defining the active space for the CASPT2//CASSCF (14e/11o) calculations.

4. Optimized Structures

(**a**)

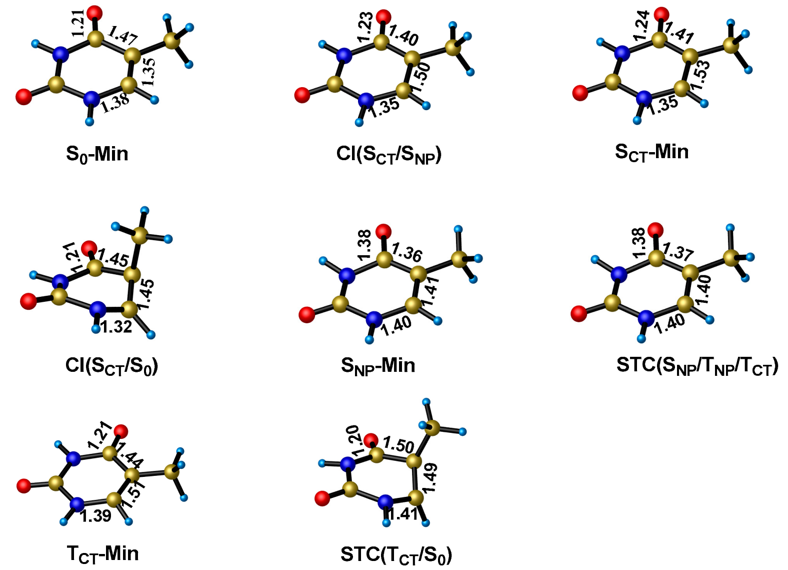

(**b**)

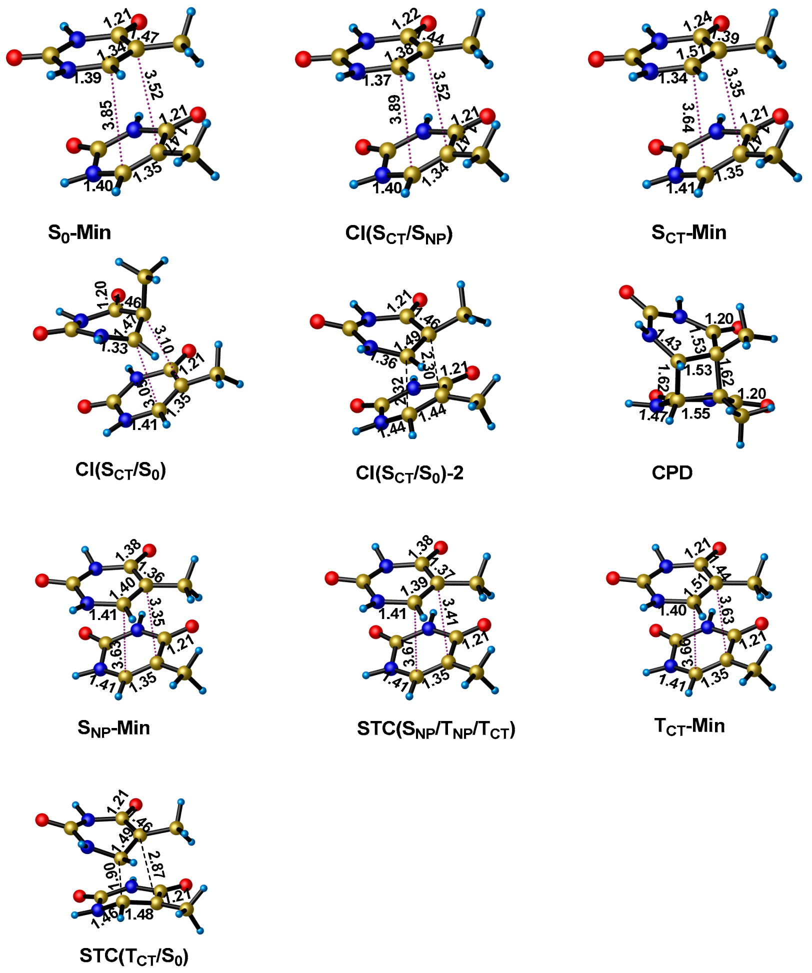

Figure S4. The structures optimized for the ground and excited states are schematically shown below: (**a**) the thymine monomer obtained at the CASSCF level of theory; (**b**) the thymine oligomer obtained at the CASSCF level of theory. Selected key bond lengths (Å) are given (see Section 7 for full Cartesian coordinates obtained at the CASSCF level of theory).

5. Tables

Table S3. Vertical excitation energies ($E_{\perp}$, nm), oscillator strengths (f), transition dipole moments (ΔD.M., Debye), and singly occupied orbitals involved in the different transitions of the thymine monomer. The values were computed with the 6-roots state-averaged CASPT2//CASSCF(14e,10o)/AMBER method.

Transitions	$E_{\perp}$	*f*	ΔD.M.	*Singly Occupied Orbitals*	
$S_0 \rightarrow S_{NP}$	255.2	7.78×10^{-4}	4.11→2.80	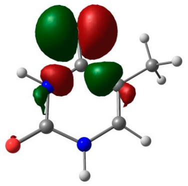	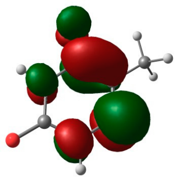
$S_0 \rightarrow S_{CT}$	253.8	0.30	4.11→5.31	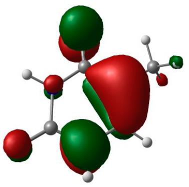	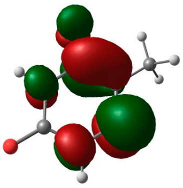

$S_0 \rightarrow S_4$	207.7	7.14×10^{-3}	4.11→1.96	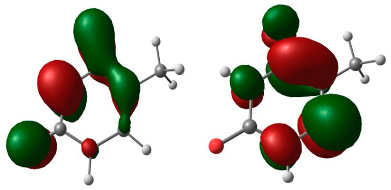
$S_0 \rightarrow S_5$	193.9	0.21	4.11→6.02	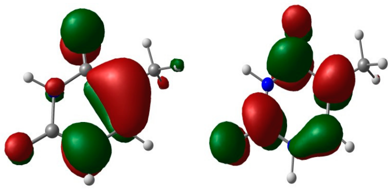
$S_0 \rightarrow S_6$	179.7	3.17×10^{-4}	4.11→3.58	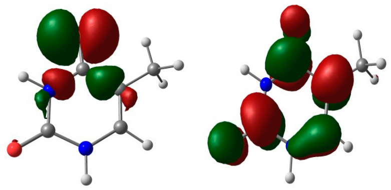

Table S4. Vertical excitation energies (E⊥, nm), oscillator strengths (f), transition dipole moments (ΔD.M., Debye), and singly occupied orbitals involved in the different transitions of the thymine oligomer. The values were computed with the 6-roots state-averaged CASPT2//CASSCF(14e,11o)/AMBER method.

Transitions	E⊥	*f*	ΔD.M.	*Singly Occupied Orbitals*
$S_0 \rightarrow S_{CT}$	257.3	0.30	7.94→10.39	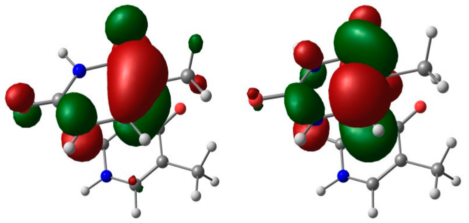
$S_0 \rightarrow S_{NP}$	256.1	5.83×10^{-4}	7.94→5.19	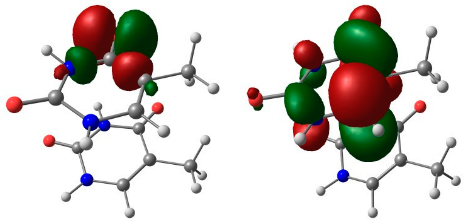
$S_0 \rightarrow S_4$	200.3	3.84×10^{-2}	7.94→5.17	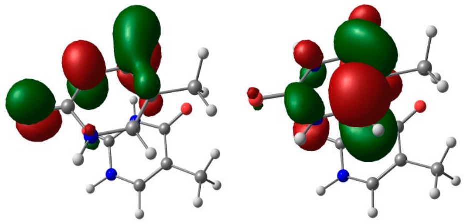
$S_0 \rightarrow S_5$	174.7	1.70×10^{-4}	7.94→5.16	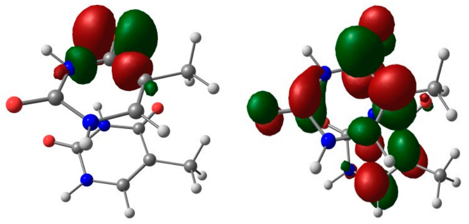

$S_0 \rightarrow S_6$	164.6	1.16×10^{-3}	7.94→7.51	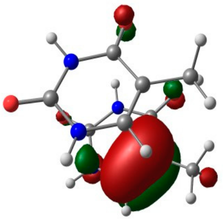	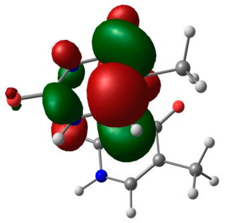

Table S5. Absolute energies (A.E., hartree), relative energies (R.E., eV/mol) and MM energies (hartree) for the optimized structures of thymine monomer along the relaxation pathway in the singlet excited state. The corresponding energy profiles are plotted in Figure 1a of the main article.

**Thymine Monomer**	**RASSCF**	**MM**	**CASPT2**	
	**A.E.**	**A.E.**	**A.E.**	**R.E.**
S_0	−451.60690	−9.66486	−452.87549	0.00
Root2	−451.42363		−452.69695	4.85
Root3($S_{NP}(^1n\pi^*)$)	−451.36081		−452.69603	4.88
Root4($S_{CT}(^1\pi\pi^*)$)	−451.34050		−452.65614	5.97
Root5	−451.32206		−452.62196	6.89
Root6	−451.28446		−452.64045	6.39
Path1−($S_{CT}(^1\pi\pi^*)$)−1				
Root1	−451.60399	−9.66021	−452.87142	0.23
Root2($S_{NP}(^1n\pi^*)$)	−451.43650		−452.70380	4.79
Root3($S_{CT}(^1\pi\pi^*)$)	−451.37030		−452.70248	4.83
Root4	−451.35999		−452.66793	5.77
Root5	−451.32786			
Root6	−451.28219			
Path1−($S_{CT}(^1\pi\pi^*)$)−2				
Root1	−451.59929	−9.66097	−452.86769	0.31
Root2($S_{NP}(^1n\pi^*)$)	−451.43944		−452.70569	4.72
Root3($S_{CT}(^1\pi\pi^*)$)	−451.37590		−452.70199	4.82
Root4	−451.36482		−452.67465	5.57
Root5	−451.32846			
Root6	−451.28521			
Path1−($S_{CT}(^1\pi\pi^*)$)−3				
Root1	−451.59016	−9.66500	−452.86383	0.31
Root2($S_{NP}(^1n\pi^*)$)	−451.43902		−452.70568	4.61
Root3($S_{CT}(^1\pi\pi^*)$)	−451.38330		−452.70105	4.74
Root4	−451.36479		−452.67768	5.37
Root5	−451.32579			
Root6	−451.28281			
Path1−($S_{CT}(^1\pi\pi^*)$)−4				
CI(S_{CT}/S_{NP})				
Root1	−451.58314	−9.66588	−452.85486	0.53
Root2($S_{NP}(^1n\pi^*)$)	−451.43635		−452.70206	4.69
Root3($S_{CT}(^1\pi\pi^*)$)	−451.38579		−452.70221	4.68
Root4	−451.36290		−452.67589	5.40
Root5	−451.32260			
Root6	−451.27847			
Path1−($S_{CT}(^1\pi\pi^*)$)−5				
Root1	−451.57946	−9.66610	−452.85207	0.60
Root2($S_{NP}(^1n\pi^*)$)	−451.43443		−452.70063	4.72
Root3($S_{CT}(^1\pi\pi^*)$)	−451.38611		−452.703354	4.65

Root4	−451.36260		−452.67525	5.41
Root5	−451.32075			
Root6	−451.27733			
Path1−($S_{CT}(^1\pi\pi^*)$)−6				
Root1	−451.57673	−9.66665	−452.84980	0.65
Root2($S_{NP}(^1n\pi^*)$)	−451.43304		−452.69947	4.74
Root3($S_{CT}(^1\pi\pi^*)$)	−451.38609		−452.70379	4.62
Root4	−451.36230		−452.67453	5.41
Root5	−451.31922			
Root6	−451.27650			
Path1−($S_{CT}(^1\pi\pi^*)$)−7				
S_{CT}−Min				
Root1	−451.57337	−9.66666	−452.84665	0.73
Root2($S_{NP}(^1n\pi^*)$)	−451.43204		−452.69849	4.76
Root3($S_{CT}(^1\pi\pi^*)$)	−451.38612		−452.70392	4.61
Root4	−451.36226		−452.67394	5.43
Root5	−451.31715			
Root6	−451.27592			
Path1−($S_{CT}(^1\pi\pi^*)$)−8				
Root1	−451.56730	−9.66635	−452.84050	0.91
Root2	−451.43134		−452.69776	4.79
Root3($S_{CT}(^1\pi\pi^*)$)	−451.38593		−452.70363	4.63
Root4	−451.36292		−452.67374	5.44
Root5	−451.31303			
Root6	−451.27594			
Path1−($S_{CT}(^1\pi\pi^*)$)−9				
Root1	−451.57363	−9.66100	−452.84769	0.86
Root2	−451.43276		−452.69894	4.90
Root3($S_{CT}(^1\pi\pi^*)$)	−451.38775		−452.70551	4.73
Root4	−451.36165		−452.67239	5.63
Root5	−451.31582			
Root6	−451.27599			
Path1−($S_{CT}(^1\pi\pi^*)$)−10				
Root1	−451.57378	−9.66063	−452.84487	0.94
Root2	−451.43308		−452.69762	4.95
Root3($S_{CT}(^1\pi\pi^*)$)	−451.38778		−452.70592	4.72
Root4	−451.36175		−452.67145	5.66
Root5	−451.31628			
Root6	−451.27627			
Path1−($S_{CT}(^1\pi\pi^*)$)−11				
Root1	−451.53565	−9.66181	−452.81144	1.82
Root2	−451.41074		−452.67901	5.42
Root3($S_{CT}(^1\pi\pi^*)$)	−451.38886		−452.70848	4.62
Root4	−451.34834		−452.64667	6.30
Root5	−451.29216			
Root6	−451.26245			
Path1−($S_{CT}(^1\pi\pi^*)$)−12				
Root1	−451.48831	−9.66580	−452.76056	3.10
Root2	−451.39185		−452.66142	5.79
Root3($S_{CT}(^1\pi\pi^*)$)	−451.38555		−452.71189	4.42
Root4	−451.33319		−452.61631	7.02
Root5	−451.25707			

Root6	−451.25482			
Path1−(S_{CT}($^1\pi\pi^*$))−13				
Root1	−451.47773	−9.66540	−452.75166	3.35
Root2(S_{CT}($^1\pi\pi^*$))	−451.39588		−452.71668	4.30
Root3	−451.38299		−452.65694	5.93
Root4	−451.32602		−452.61021	7.20
Root5	−451.25046			
Root6	−451.24254			
Path1−(S_{CT}($^1\pi\pi^*$))−14				
Root1	−451.46954	−9.66585	−452.74374	3.55
Root2(S_{CT}($^1\pi\pi^*$))	−451.40352		−452.72263	4.13
Root3	−451.35884		−452.64699	6.19
Root4	−451.31241		−452.59753	7.53
Root5	−451.25632			
Root6	−451.24428			
Path1−(S_{CT}($^1\pi\pi^*$))−15				
Root1	−451.45745	−9.66572	−452.73344	3.84
Root2(S_{CT}($^1\pi\pi^*$))	−451.40987		−452.72371	4.10
Root3	−451.33168		−452.62556	6.77
Root4	−451.29208		−452.58256	7.94
Root5	−451.25219			
Root6	−451.23991			
Path1−(S_{CT}($^1\pi\pi^*$))−16 CI(S_{CT}/S_0)				
Root1	−451.45399	−9.66557	−452.73142	3.90
Root2(S_{CT}($^1\pi\pi^*$))	−451.41001		−452.72349	4.11
Root3	−451.32105		−452.61713	7.01
Root4	−451.28365		−452.57704	8.10
Root5	−451.25319			
Root6	−451.24065			

Table S6. Absolute energies (A.E., hartree), relative energies (R.E., eV/mol) and MM energies (hartree) for the optimized structures of thymine monomer along the relaxation pathway in the triplet excited state. The corresponding energy profiles are plotted in Figure 1b of the main article.

Thymine Monomer	RASSCF	MM	CASPT2	
	A.E.	A.E.	A.E.	R.E.
Path2−S_{NP}($^1n\pi^*$)−1				
Root1	−451.57043	−9.66756	−452.84481	0.76
Root2(S_{NP}($^1n\pi^*$))	−451.46102		−452.72115	4.12
Root3	−451.38826		−452.68130	5.21
Root4	−451.34460		−452.68494	5.11
Root5	−451.33734			
Root6	−451.27273			
Path2−S_{NP}($^1n\pi^*$)−2				
Root1	−451.56125	−9.66712	−452.83789	0.96
Root2(S_{NP}($^1n\pi^*$))	−451.46244		−452.72307	4.08
Root3	−451.39159		−452.68583	5.09
Root4	−451.34142		−452.68507	5.11
Root5	−451.33653			
Root6	−451.27100			
Path2−S_{NP}($^1n\pi^*$)−3				
Root1	−451.56028	−9.66714	−452.83688	0.98

Root2(S_NP(¹nπ*))	−451.46240		−452.72287	4.09
Root3	−451.39196		−452.68609	5.09
Root4	−451.34103		−452.68449	5.13
Root5	−451.33613			
Root6	−451.27034			
Path2−S_NP(¹nπ*)−4				
S_NP−Min				
Root1	−451.55907	−9.66850	−452.83465	1.01
Root2(S_NP(¹nπ*))	−451.46273		−452.72283	4.05
Root3	−451.38840		−452.68241	5.15
Root4	−451.33903		−452.67316	5.40
Root5	−451.33771			
Root6	−451.27032			
Path2−T_CT(³ππ*)−1				
Root1	−451.56531	−9.66481	−452.84085	0.94
Root2	−451.46237		−452.72288	4.15
Root3	−451.39271		−452.68541	5.17
Root4	−451.34591		−452.68862	5.08
Root5	−451.33713			
Root6	−451.27373			
Root1(T_CT(³ππ*))	−451.47932		−452.74414	3.57
Root2	−451.46709		−452.72663	4.05
Root3	−451.40190		−452.69356	4.95
Root4	−451.34527		−452.62397	6.84
Root5	−451.32838			
Root6	−451.30423			
Path2−T_CT(³ππ*)−2				
Root1	−451.57495	−9.66441	−452.84897	0.73
Root2	−451.46080		−452.72189	4.19
Root3	−451.39398		−452.68594	5.17
Root4	−451.35527		−452.69494	4.92
Root5	−451.33871			
Root6	−451.27932			
Root1(T_CT(³ππ*))	−451.48297		−452.75073	3.40
Root2	−451.46635		−452.72685	4.05
Root3	−451.40841		−452.69775	4.84
Root4	−451.34815		−452.62783	6.75
Root5	−451.33281			
Root6	−451.31186			
Path2−T_CT(³ππ*)−3				
Root1	−451.58846	−9.66467	−452.85914	0.45
Root2	−451.45025		−452.71398	4.40
Root3	−451.38735		−452.68635	5.15
Root4	−451.36507		−452.69608	4.88
Root5	−451.33455			
Root6	−451.28341			
Root1(T_CT(³ππ*))	−451.48893		−452.76065	3.13
Root2	−451.45469		−452.71893	4.26
Root3	−451.40894		−452.69500	4.91
Root4	−451.34377		−452.62701	6.76
Root5	−451.33898			
Root6	−451.31844			

Path2−T_{CT}($^3\pi\pi^*$)−4				
Root1	−451.58917	−9.66559	−452.85770	0.46
Root2	−451.43316		−452.69994	4.75
Root3	−451.37730		−452.69433	4.90
Root4	−451.36083		−452.67777	5.36
Root5	−451.32234			
Root6	−451.27737			
Root1(T_{CT}($^3\pi\pi^*$))	−451.49118		−452.76272	3.04
Root2	−451.43717		−452.70565	4.60
Root3	−451.39582		−452.68381	5.19
Root4	−451.33881		−452.63887	6.41
Root5	−451.33211			
Root6	−451.31310			
Path2−T_{CT}($^3\pi\pi^*$)−5				
Root1	−451.58641	−9.66549	−452.85541	0.52
Root2	−451.43024		−452.69778	4.81
Root3	−451.37586		−452.69481	4.89
Root4	−451.36039		−452.67648	5.39
Root5	−451.32027			
Root6	−451.27643			
Root1(T_{CT}($^3\pi\pi^*$))	−451.49174		−452.76363	3.02
Root2	−451.43418		−452.70347	4.66
Root3	−451.39395		−452.68228	5.24
Root4	−451.33721		−452.63842	6.43
Root5	−451.33031			
Root6	−451.31116			
Path2−T_{CT}($^3\pi\pi^*$)−6				
Root1	−451.58305	−9.66553	−452.85201	0.62
Root2	−451.42953		−452.69713	4.83
Root3	−451.37539		−452.69358	4.93
Root4	−451.36117		−452.67804	5.35
Root5	−451.31859			
Root6	−451.27678			
Root1(T_{CT}($^3\pi\pi^*$))	−451.49332		−452.76452	3.00
Root2	−451.43328		−452.70242	4.69
Root3	−451.39282		−452.68074	5.28
Root4	−451.33444		−452.63584	6.50
Root5	−451.32876			
Root6	−451.30799			
Path2−T_{CT}($^3\pi\pi^*$)−7				
T_{CT}−Min				
Root1	−451.57191	−9.66443	−452.84023	0.97
Root2	−451.42834		−452.69620	4.89
Root3	−451.37269		−452.68459	5.20
Root4	−451.36235		−452.68388	5.22
Root5	−451.31154			
Root6	−451.27901			
Root1(T_{CT}($^3\pi\pi^*$))	−451.49766		−452.76603	2.99
Root2	−451.43042		−452.69979	4.79
Root3	−451.38897		−452.67677	5.41
Root4	−451.32546		−452.62477	6.83
Root5	−451.32083			

Root6	−451.29932			
Path2−T_{CT}($^3\pi\pi^*$)−8				
Root1	−451.53031	−9.67054	−452.80549	1.75
Root2	−451.38635		−452.68507	5.02
Root3	−451.35621		−452.67946	5.17
Root4	−451.33969		−452.64815	6.03
Root5	−451.31312			
Root6	−451.28053			
Root1(T_{CT}($^3\pi\pi^*$))	−451.49179		−452.75866	3.02
Root2	−451.38925		−452.66557	5.55
Root3	−451.35393		−452.64149	6.21
Root4	−451.31308		−452.61803	6.85
Root5	−451.30463			
Root6	−451.30009			
Path2−T_{CT}($^3\pi\pi^*$)−9				
Root1	−451.51413	−9.67059	−452.79137	2.13
Root2	−451.35525		−452.68830	4.93
Root3	−451.32166		−452.63598	6.36
Root4	−451.30857		−452.60834	7.11
Root5	−451.28767			
Root6	−451.26007			
Root1(T_{CT}($^3\pi\pi^*$))	−451.49033		−452.75646	3.08
Root2	−451.37292		−452.65188	5.92
Root3	−451.33774		−452.62501	6.65
Root4	−451.30867		−452.60028	7.33
Root5	−451.29680			
Root6	−451.29194			
Path2−T_{CT}($^3\pi\pi^*$)−10				
Root1	−451.49536	−9.67442	−452.76326	2.79
Root2	−451.39289			
Root3	−451.33982			
Root4	−451.32774			
Root5	−451.27673			
Root6	−451.27461			
Root1(T_{CT}($^3\pi\pi^*$))	−451.49052		−452.75202	3.09
Root2	−451.38254			
Root3	−451.33301			
Root4	−451.30623			
Root5	−451.29205			
Root6	−451.27201			
Path2−T_{CT}($^3\pi\pi^*$)−11				
Root1	−451.49091	−9.67452	−452.75960	2.89
Root2	−451.38593			
Root3	−451.33287			
Root4	−451.31946			
Root5	−451.27905			
Root6	−451.27217			
Root1(T_{CT}($^3\pi\pi^*$))	−451.48867		−452.75074	3.13
Root2	−451.37062			
Root3	−451.32352			
Root4	−451.30813			
Root5	−451.29341			

Root6	−451.27418			
Path2−T_{CT}($^{3}\pi\pi^{*}$)−12				
Root1	−451.48613	−9.67401	−452.75461	3.04
Root2	−451.38064			
Root3	−451.32590			
Root4	−451.31205			
Root5	−451.27914			
Root6	−451.26829			
Root1(T_{CT}($^{3}\pi\pi^{*}$))	−451.48646		−452.74843	3.20
Root2	−451.36100			
Root3	−451.31466			
Root4	−451.30803			
Root5	−451.29275			
Root6	−451.27330			
Path2−T_{CT}($^{3}\pi\pi^{*}$)−13 STC(T_{CT}/S_0)				
Root1	−451.486437	−9.67496	−452.75396	3.03
Root2	−451.36666			
Root3	−451.31313			
Root4	−451.30212			
Root5	−451.28968			
Root6	−451.27433			
Root1(T_{CT}($^{3}\pi\pi^{*}$))	−451.48668		−452.74745	3.20
Root2	−451.34766			
Root3	−451.31668			
Root4	−451.30270			
Root5	−451.29876			
Root6	−451.27928			

Table S7. Absolute energies (A.E., hartree), relative energies (R.E., eV/mol) and MM energies (hartree) for the optimized structures of thymine oligomer along the relaxation pathway in the singlet excited state. The corresponding energy profiles are plotted in the right of Figure 2a in the main article.

**Thymine Oligomer**	**RASSCF**	**MM**	**CASPT2**	
	**A.E.**	**A.E.**	**A.E.**	**R.E.**
S_0	−903.16987	−29.98257	−905.75157	0.00
Root2(S_{NP}($^{1}n\pi^{*}$))	−902.98646		−905.57357	4.84
Root3(S_{CT}($^{1}\pi\pi^{*}$))	−902.91004		−905.57437	4.82
Root4	−902.89802		−905.52396	6.19
Root5	−902.87463		−905.49057	7.10
Root6	−902.87093		−905.47462	7.53
Path3−(S_{CT}($^{1}\pi\pi^{*}$))−1				
Root1	−903.16831	−29.97864	−905.75118	0.11
Root2(S_{NP}($^{1}n\pi^{*}$))	−902.99363		−905.57913	4.79
Root3(S_{CT}($^{1}\pi\pi^{*}$))	−902.91749		−905.58037	4.76
Root4	−902.90783		−905.53182	6.08
Root5	−902.88193			
Root6	−902.87776			
Path3−(S_{CT}($^{1}\pi\pi^{*}$))−2 CI(S_{CT}/S_{NP})				
Root1	−903.16602	−29.97841	−905.74987	0.15
Root2(S_{NP}($^{1}n\pi^{*}$))	−902.99692		−905.58165	4.73
Root3(S_{CT}($^{1}\pi\pi^{*}$))	−902.92147		−905.58087	4.75

Root4	−902.91261		−905.53837	5.91
Root5	−902.88509			
Root6	−902.88109			
Path3−(S_{CT}($^1\pi\pi^*$))−3				
S_{CT}−Min				
Root1	−903.12917	−29.97784	−905.72385	0.88
Root2(S_{NP}($^1n\pi^*$))	−902.99166		−905.57874	4.83
Root3(S_{CT}($^1\pi\pi^*$))	−902.93884		−905.58241	4.73
Root4	−902.91592		−905.55440	5.49
Root5	−902.89305			
Root6	−902.87577			
Path3−(S_{CT}($^1\pi\pi^*$))−4				
Root1	−903.10404	−29.98042	−905.70141	1.42
Root2(S_{NP}($^1n\pi^*$))	−902.97848		−905.56948	5.01
Root3(S_{CT}($^1\pi\pi^*$))	−902.93534		−905.58851	4.49
Root4	−902.90938			
Root5	−902.89562			
Root6	−902.85628			
Path3−(S_{CT}($^1\pi\pi^*$))−5				
Root1	−903.08919	−29.97941	−905.68912	1.78
Root2(S_{NP}($^1n\pi^*$))	−902.96759		−905.56298	5.21
Root3(S_{CT}($^1\pi\pi^*$))	−902.93598		−905.59271	4.40
Root4	−902.90253			
Root5	−902.89360			
Root6	−902.84523			
Path3−(S_{CT}($^1\pi\pi^*$))−6				
Root1	−903.07060	−29.97984	−905.67474	2.16
Root2(S_{NP}($^1n\pi^*$))	−902.95450		−905.56267	5.21
Root3(S_{CT}($^1\pi\pi^*$))	−902.93779		−905.59541	4.32
Root4	−902.89462			
Root5	−902.88983			
Root6	−902.83057			
Path3−(S_{CT}($^1\pi\pi^*$))−7				
Root1	−903.05055	−29.97665	−905.65857	2.69
Root2(S_{CT}($^1\pi\pi^*$))	−902.94722		−905.60333	4.19
Root3	−902.92934		−905.54456	5.79
Root4	−902.89054			
Root5	−902.87824			
Root6	−902.80370			
Path3−(S_{CT}($^1\pi\pi^*$))−8				
Root1	−903.03353	−29.97675	−905.64613	3.02
Root2(S_{CT}($^1\pi\pi^*$))	−902.95759		−905.60454	4.15
Root3	−902.88503		−905.51553	6.58
Root4	−902.86467			
Root5	−902.80345			
Root6	−902.79724			
Path3−(S_{CT}($^1\pi\pi^*$))−9				
Root1	−903.02520	−29.97558	−905.63763	3.29
Root2(S_{CT}($^1\pi\pi^*$))	−902.95937		−905.60327	4.22
Root3	−902.87963		−905.50855	6.80
Root4	−902.85448			
Root5	−902.79661			

Root6	−902.79524			
Path3−($S_{CT}(^1\pi\pi^*)$)−10				
Root1	−903.01734		−905.62251	3.73
Root2($S_{CT}(^1\pi\pi^*)$)	−902.94934		−905.60195	4.29
Root3	−902.88694		−905.51283	6.71
Root4	−902.87265			
Root5	−902.84568			
Root6	−902.79538			
Path3−($S_{CT}(^1\pi\pi^*)$)−11				
Root1	−903.00616		−905.60994	4.09
Root2($S_{NP}(^1n\pi^*)$)	−902.95002		−905.59944	4.37
Root3($S_{CT}(^1\pi\pi^*)$)	−902.86801		−905.50090	7.05
Root4	−902.86264			
Root5	−902.82944			
Root6	−902.78936			
Path3−($S_{CT}(^1\pi\pi^*)$)−12				
CI(S_{CT}/S_0)				
Root1	−902.99904	−29.97516	−905.60384	4.22
Root2 ($S_{CT}(^1\pi\pi^*)$)	−902.94832		−905.59755	4.39
Root3	−902.85842		−905.49310	7.23
Root4	−902.85527			
Root5	−902.81910			
Root6	−902.78428			

Table S8. Absolute energies (A.E., hartree), relative energies (R.E., eV/mol) and MM energies (hartree) for the optimized structures of thymine oligomer along the relaxation pathway in the singlet excited state. The corresponding energy profiles are plotted in the left of Figure 2a in the main article.

Thymine oligomer	RASSCF	MM	CASPT2	
	A.E.	A.E.	A.E.	R.E.
Path4−($S_{CT}(^1\pi\pi^*)$)−1				
Root1	−903.12917	−29.97784	−905.72385	0.88
Root2	−902.99166		−905.57874	4.83
Root3($S_{CT}(^1\pi\pi^*)$)	−902.93884		−905.58241	4.73
Root4	−902.91592		−905.55440	5.49
Root5	−902.89305			
Root6	−902.87577			
Path4−($S_{CT}(^1\pi\pi^*)$)−2				
Root1	−903.09472	−29.97599	−905.69336	1.76
Root2	−902.96973		−905.56314	5.30
Root3($S_{CT}(^1\pi\pi^*)$)	−902.93575		−905.58842	4.61
Root4	−902.90403		−905.52842	6.25
Root5	−902.89129			
Root6	−902.85252			
Path4−($S_{CT}(^1\pi\pi^*)$)−3				
Root1	−903.08786	−29.97601	−905.68741	1.92
Root2	−902.96498		−905.56107	5.36
Root3($S_{CT}(^1\pi\pi^*)$)	−902.93596		−905.59017	4.57
Root4	−902.90115		−905.52195	6.42
Root5	−902.89067			
Root6	−902.84748			
Path4−($S_{CT}(^1\pi\pi^*)$)−4				
Root1	−903.06827	−29.97529	−905.67092	2.39

Root2	−902.95137		−905.56466	5.28
Root3(S_{CT}($^1\pi\pi^*$))	−902.93762		−905.59228	4.53
Root4	−902.89280		−905.50991	6.77
Root5	−902.88754			
Root6	−902.83442			
Path4−(S_{CT}($^1\pi\pi^*$))−5				
Root1	−903.04618	−29.97364	−905.65445	2.88
Root2(S_{CT}($^1\pi\pi^*$))	−902.94566		−905.60097	4.34
Root3	−902.92378		−905.53844	6.04
Root4	−902.88698		−905.51268	6.74
Root5	−902.87455			
Root6	−902.80600			
Path4−(S_{CT}($^1\pi\pi^*$))−6				
Root1	−903.02460	−29.97476	−905.64003	3.24
Root2(S_{CT}($^1\pi\pi^*$))	−902.95406		−905.60266	4.26
Root3	−902.88733		−905.52426	6.39
Root4	−902.84979		−905.49499	7.19
Root5	−902.80010			
Root6	−902.79488			
Path4−(S_{CT}($^1\pi\pi^*$))−7				
Root1	−903.00654	−29.98014	−905.63488	3.24
Root2(S_{CT}($^1\pi\pi^*$))	−902.98266		−905.62487	3.51
Root3	−902.87222		−905.55016	5.54
Root4	−902.83084		−905.49520	7.04
Root5	−902.79157			
Root6	−902.77666			
Path4−(S_{CT}($^1\pi\pi^*$))−8				
CI(S_{CT}/S_0)−2				
Root1	−902.99919	−29.981189	−905.62979	3.35
Root2(S_{CT}($^1\pi\pi^*$))	−902.98368		−905.62824	3.39
Root3	−902.86393		−905.54427	5.67
Root4	−902.82068		−905.48924	7.17
Root5	−902.78273			
Root6	−902.76717			
Path4−S_0−9				
Root1	−903.03472	−29.97482	−905.64456	3.12
Root2	−902.89373		−905.55186	5.64
Root3	−902.88662		−905.51989	6.51
Root4	−902.87074			
Root5	−902.83130			
Root6	−902.81034			
Path4−S_0−10				
Root1	−903.03949	−29.97326	−905.64877	3.05
Root2	−902.88427		−905.53164	6.23
Root3	−902.87787		−905.53634	6.10
Root4	−902.86589			
Root5	−902.822766			
Root6	−902.80912			
Path4−S_0−11				
Root1	−903.05049	−29.97326	−905.66130	2.70
Root2	−902.88781		−905.51901	6.58
Root3	−902.86297		−905.57343	5.10

Root4	−902.84343			
Root5	−902.82251			
Root6	−902.81115			
Path4−S_0−12				
Root1	−903.06502	−29.97230	−905.67741	2.29
Root2	−902.89306		−905.52466	6.45
Root3	−902.85173		−905.57267	5.14
Root4	−902.81959			
Root5	−902.81163			
Root6	−902.79464			
Path4−S_0−13				
Root1	−903.07575	−29.97199	−905.69212	1.90
Root2	−902.89949		−905.53499	6.18
Root3	−902.83938		−905.56346	5.40
Root4	−902.82004			
Root5	−902.81005			
Root6	−902.78575			
Path4−S_0−14				
Root1	−903.07918	−29.97167	−905.69696	1.78
Root2	−902.90040		−905.53714	6.13
Root3	−902.83596		−905.56161	5.46
Root4	−902.81662			
Root5	−902.80689			
Root6	−902.78475			
Path4−S_0−15				
Root1	−903.08516	−29.97155	−905.70583	1.54
Root2	−902.90172		−905.54110	6.02
Root3	−902.82583		−905.55507	5.64
Root4	−902.81086			
Root5	−902.79778			
Root6	−902.78208			
Path4−S_0−16				
Root1	−903.08961	−29.97138	−905.71403	1.32
Root2	−902.90196		−905.54494	5.92
Root3	−902.81628		−905.54931	5.80
Root4	−902.80445			
Root5	−902.78571			
Root6	−902.77681			
Path4−S_0−17				
Root1	−903.09254	−29.97136	−905.72125	1.12
Root2	−902.90111		−905.54839	5.83
Root3	−902.80772		−905.54559	5.90
Root4	−902.79862			
Root5	−902.77436			
Root6	−902.77168			
Path4−S_0−18				
Root1	−903.10511	−29.97175	−905.73247	0.81
Root2	−902.90736		−905.55083	5.75
Root3	−902.89787		−905.55960	5.51
Root4	−902.77442			
Root5	−902.75487			
Root6	−902.74138			

Path4–S_0–19				
Root1	−903.10516	−29.971935	−905.73832	0.65
Root2	−902.90382		−905.55991	5.50
Root3	−902.87505		−905.52179	6.54
Root4	−902.80329			
Root5	−902.77103			
Root6	−902.76235			
Path4–S_0–20				
Root1	−903.10940	−29.97247	−905.74297	0.50
Root2	−902.90707		−905.56284	5.41
Root3	−902.87953		−905.52585	6.41
Root4	−902.80585			
Root5	−902.77314			
Root6	−902.76231			
Path4–S_0–21				
CPD				
Root1	−903.11574	−29.972826	−905.74927	0.32
Root2	−902.91127		−905.56651	5.30
Root3	−902.88565		−905.53111	6.263
Root4	−902.80844			
Root5	−902.77451			
Root6	−902.76228			

Table S9. Absolute energies (A.E., hartree), relative energies (R.E., eV/mol) and MM energies (hartree) for the optimized structures of thymine oligomer along the relaxation pathway in the triplet excited state. The corresponding energy profiles are plotted in Figure 2b in the main article.

**Thymine oligomer**	**RASSCF**	**MM**	**CASPT2**	
	**A.E.**	**A.E.**	**A.E.**	**R.E.**
Path5–(S_{NP}($^1n\pi^*$))–1				
Root1	−903.12883	−29.98393	−905.72358	0.72
Root2(S_{NP}($^1n\pi^*$))	−902.99588		−905.58201	4.57
Root3	−902.93988		−905.57780	4.69
Root4	−902.91776		−905.56224	5.11
Root5	−902.89325			
Root6	−902.87890			
Path5–(S_{NP}($^1n\pi^*$))–2				
Root1	−903.12495	−29.98403	−905.72081	0.79
Root2(S_{NP}($^1n\pi^*$))	−903.00788		−905.59149	4.31
Root3	−902.94520		−905.56632	5.00
Root4	−902.91645		−905.57762	4.69
Root5	−902.89107			
Root6	−902.88770			
Path5–(S_{NP}($^1n\pi^*$))–3				
Root1	−903.11703	−29.98409	−905.71395	0.98
Root2(S_{NP}($^1n\pi\pi$))	−903.01792		−905.59919	4.10
Root3	−902.94912		−905.56311	5.08
Root4	−902.90510			
Root5	−902.89458			
Root6	−902.88566			
Path5–(S_{NP}($^1n\pi^*$))–4				
Root1	−903.11372	−29.98427	−905.71075	1.06
Root2(S_{NP}($^1n\pi^*$))	−903.02088		−905.60136	4.04

Root3	−902.94826		−905.56172	5.11
Root4	−902.89739			
Root5	−902.89715			
Root6	−902.88234			
Path5−(S_{NP}(^{1}nπ*))−5				
S_{NP}−Min				
Root1	−903.11770	−29.98479	−905.712829	0.99
Root2(S_{NP}(^{1}nπ*))	−903.02403		−905.60296	3.98
Root3	−902.94286		−905.55535	5.27
Root4	−902.90007			
Root5	−902.88978			
Root6	−902.87953			
Path5−(S_{NP}(^{1}nπ*))−6				
Root1	−903.11812	−29.98437	−905.71173	1.03
Root2(S_{NP}(^{1}nπ*))	−903.02488		−905.60275	4.00
Root3	−902.93861		−905.54904	5.46
Root4	−902.90188			
Root5	−902.88640			
Root6	−902.87815			
Path5−(S_{CT}(^{1}ππ*))−7				
STC(S_{NP}/T_{NP}/T_{CT})				
Root1	−903.11797	−29.98430	−905.71116	1.05
Root2(S_{NP}(^{1}nπ*))	−903.02506		−905.60262	4.00
Root3	−902.93772		−905.54745	5.50
Root4	−902.90273			
Root5	−902.88586			
Root6	−902.87788			
Root1(T_{CT}(^{3}ππ*))	−903.03336	−29.98430	−905.61275	3.73
Root2(T_{NP}(^{3}nπ*))	−903.02921		−905.60584	3.91
Root3	−902.96872		−905.56587	5.00
Root4	−902.95475			
Root5	−902.90591			
Root6	−902.88577			
Path6−(T_{CT}(^{3}ππ*))−1				
Root1	−903.12550	−29.98183	−905.72050	0.86
Root2	−902.94147		−905.55773	5.29
Root3	−902.90292		−905.56710	5.03
Root4	−902.88366			
Root5	−902.83045			
Root6	−902.81909			
Root1(T_{CT}(^{3}ππ*))	−903.03210		−905.61979	3.60
Root2	−902.97756		−905.57893	4.71
Root3	−902.96137		−905.57228	4.89
Root4	−902.88969			
Root5	−902.88496			
Root6	−902.86655			
Path6−(T_{CT}(^{3}ππ*))−2				
Root1	−903.13026	−29.98178	−905.72433	0.76
Root2	−902.94286		−905.55823	5.28
Root3	−902.90857		−905.57116	4.93
Root4	−902.88667			
Root5	−902.83382			

Root6	−902.82225			
Root1(T_{CT}($^3\pi\pi^*$))	−903.03478		−905.62377	3.49
Root2	−902.98219		−905.58302	4.60
Root3	−902.96497		−905.57510	4.82
Root4	−902.89293			
Root5	−902.88745			
Root6	−902.86919			
Path6−(T_{CT}($^3\pi\pi^*$))−3				
Root1	−903.14364	−29.98170	−905.73096	0.58
Root2	−903.01782		−905.59522	4.27
Root3	−902.94284		−905.54986	5.51
Root4	−902.91208			
Root5	−902.89909			
Root6	−902.89053			
Root1(T_{CT}($^3\pi\pi^*$))	−903.04229		−905.63403	3.22
Root2	−902.99610		−905.59466	4.29
Root3	−902.96833		−905.57733	4.76
Root4	−902.90409			
Root5	−902.89476			
Root6	−902.87324			
Path6−(T_{CT}($^3\pi\pi^*$))−4				
Root1	−903.14950	−29.98179	−905.73364	0.50
Root2	−902.99017		−905.57439	4.84
Root3	−902.92460		−905.56034	5.22
Root4	−902.91408			
Root5	−902.89560			
Root6	−902.88063			
Root1(T_{CT}($^3\pi\pi^*$))	−903.04958		−905.63876	3.09
Root2	−903.00176		−905.59707	4.22
Root3	−902.94862		−905.56405	5.12
Root4	−902.90920			
Root5	−902.90209			
Root6	−902.86660			
Path6−(T_{CT}($^3\pi\pi^*$))−5				
Root1	−903.13566	−29.98074	−905.72059	0.89
Root2	−902.98396		−905.57050	4.97
Root3	−902.92308		−905.55770	5.32
Root4	−902.91533			
Root5	−902.90158			
Root6	−902.87591			
Root1(T_{CT}($^3\pi\pi^*$))	−903.05459		−905.64245	3.01
Root2	−902.98856		−905.58432	4.60
Root3	−902.94088		−905.55899	5.28
Root4	−902.90830			
Root5	−902.89673			
Root6	−902.86126			
Path6−(T_{CT}($^3\pi\pi^*$))−6				
T_{CT}−Min				
Root1	−903.12731	−29.97983	−905.71961	0.944
Root2	−902.93272		−905.59032	4.46
Root3	−902.91311		−905.54019	5.82
Root4	−902.90357		−905.51483	6.51

Root5	−902.84281			
Root6	−902.83093			
Root1(T_{CT}($^3\pi\pi^*$))	−903.05411		−905.64383	3.00
Root2	−902.98252		−905.57697	4.82
Root3	−902.93467		−905.55573	5.40
Root4	−902.90867		−905.50637	6.74
Root5	−902.88852			
Root6	−902.85695			
Path6−(T_{CT}($^3\pi\pi^*$))−7				
Root1	−903.10502	−29.98025	−905.70322	1.37
Root2	−902.92987		−905.59035	4.44
Root3	−902.90520		−905.53264	6.02
Root4	−902.89889		−905.53335	6.00
Root5	−902.84111			
Root6	−902.82234			
Root1(T_{CT}($^3\pi\pi^*$))	−903.04755		−905.64293	3.01
Root2	−902.96729		−905.56862	5.04
Root3	−902.91388		−905.54454	5.69
Root4	−902.90726		−905.51095	6.61
Root5	−902.86870			
Root6	−902.84287			
Path6−(T_{CT}($^3\pi\pi^*$))−8				
Root1	−903.08070	−29.97777	−905.68577	1.92
Root2	−902.92975		−905.58110	4.76
Root3	−902.90623		−905.56533	5.19
Root4	−902.87979		−905.52611	6.26
Root5	−902.82954			
Root6	−902.81233			
Root1(T_{CT}($^3\pi\pi^*$))	−903.03927		−905.64099	3.13
Root2	−902.95258		−905.56268	5.27
Root3	−902.90321		−905.51284	6.62
Root4	−902.89242		−905.53190	6.10
Root5	−902.84683			
Root6	−902.82479			
Path6−(T_{CT}($^3\pi\pi^*$))−9				
Root1	−903.05804	−29.97465	−905.67272	2.36
Root2	−902.95553		−905.59258	4.54
Root3	−902.91447		−905.60075	4.31
Root4	−902.87226		−905.52395	6.40
Root5	−902.82810453			
Root6	−902.81508852			
Root1(T_{CT}($^3\pi\pi^*$))	−903.03461		−905.64519	3.11
Root2	−902.94883		−905.57161	5.11
Root3	−902.90517		−905.53041	6.23
Root4	−902.88337		−905.52251	6.44
Root5	−902.81569			
Root6	−902.81103			
Path6−(T_{CT}($^3\pi\pi^*$))−10				
STC(T_{CT}/S_0)				
Root1	−903.04387	−29.97365	−905.66398	2.62
Root2	−902.92402		−905.59997	4.36
Root3	−902.89995		−905.60210	4.30

Root4	−902.88308		−905.53816	6.04
Root5	−902.82477			
Root6	−902.80177			
Root1(T_{CT}($^3\pi\pi^*$))	−903.04810		−905.65828	2.78
Root2	−902.88767		−905.53421	6.15
Root3	−902.87723		−905.55138	5.69
Root4	−902.86441		−905.54919	5.74
Root5	−902.81743			
Root6	−902.80801			
Path6–S_0–1				
Root1	−903.05068	−29.97294	−905.66719	2.55
Root2	−902.93639		−905.56883	5.23
Root3	−902.88582		−905.58870	4.69
Root4	−902.88003		−905.55008	5.74
Root5	−902.85611			
Root6	−902.84803			
Path6–S_0–2				
Root1	−903.05897	−29.97291	−905.67495	2.34
Root2	−902.93121		−905.55779	5.53
Root3	−902.87982		−905.52851	6.33
Root4	−902.85145		−905.57852	4.97
Root5	−902.83640			
Root6	−902.82041			
Path6–S_0–3				
Root1	−903.05334	−29.97349	−905.68683	2.00
Root2	−902.85082		−905.53151	6.23
Root3	−902.84314		−905.55263	5.66
Root4	−902.82805		−905.51568	6.66
Root5	−902.81320			
Root6	−902.77767			
Path6–S_0–4				
Root1	−903.05966	−29.97421	−905.69629	1.73
Root2	−902.83482		−905.49803	7.12
Root3	−902.82352		−905.54257	5.91
Root4	−902.81267		−905.51792	6.58
Root5	−902.78897			
Root6	−902.78062			
Path6–S_0–5				
Root1	−903.06598	−29.97492	−905.70542	1.46
Root2	−902.83750		−905.49594	7.16
Root3	−902.81059		−905.53436	6.11
Root4	−902.79778		−905.51236	6.71
Root5	−902.77768			
Root6	−902.77076			
Path6–S_0–6				
Root1	−903.08177	−29.97504	−905.71738	1.13
Root2	−902.88954		−905.54427	5.84
Root3	−902.78874		−905.53828	6.00
Root4	−902.78206			
Root5	−902.76536			
Root6	−902.72329			
Path6–S_0–7				

Root1	−903.09503	−29.97534	−905.72688	0.86
Root2	−902.88247		−905.54447	5.83
Root3	−902.80581		−905.51373	6.66
Root4	−902.79367			
Root5	−902.75957			
Root6	−902.72490			
Path6–S_0–8				
Root1	−903.10972	−29.97532	−905.73885	0.54
Root2	−902.89419		−905.55134	5.64
Root3	−902.81788		−905.52129	6.46
Root4	−902.80336			
Root5	−902.75779			
Root6	−902.73738			
Path6–S_0–9 CPD				
Root1	−903.12135	−29.97254	−905.75183	0.26
Root2	−902.92369		−905.56391	5.37
Root3	−902.80726		−905.52105	6.54
Root4	−902.78247			
Root5	−902.74760			
Root6	−902.73906			

6. Cartesian Coordinates

6.1. Thymine Monomer

**S_0–Min**			
N	0.185476	−1.653287	0.000000
C	−1.060339	−1.052397	0.000000
H	−1.897559	−1.734286	0.000000
C	−1.218752	0.286416	0.000000
C	−2.566981	0.955575	0.000000
H	−3.146799	0.695232	0.888980
H	−2.441747	2.037106	0.000000
H	−3.146799	0.695232	−0.888980
C	0.000000	1.106790	0.000000
O	0.019631	2.313932	0.000000
N	1.191679	0.406884	0.000000
H	2.034858	0.944533	0.000000
C	1.352569	−0.957175	0.000000
O	2.436428	−1.476921	0.000000
H	0.270500	−2.644337	0.000000
CI(S_{CT}/S_{NP})			
N	1.116523	−1.240110	0.006340
C	−0.195887	−1.559472	−0.005120
H	−0.505145	−2.570082	0.017820
C	−1.175638	−0.426493	−0.022650
C	−2.638108	−0.725675	0.005280
H	−2.947987	−1.298676	0.889810
H	−3.177719	0.213024	0.012560
H	−2.948337	−1.303306	−0.871620
C	−0.673780	0.882178	−0.003330
O	−1.341462	1.921237	0.007710

N	0.730810	1.037060	−0.007110
H	1.096878	1.961900	0.001720
C	1.644551	0.045961	0.007430
O	2.839591	0.215813	−0.000700
H	1.799114	−1.976899	0.009380
		S_{CT}−Min	
N	1.119771	−1.238058	0.032999
C	−0.181598	−1.596741	−0.058651
H	−0.456726	−2.620651	0.094479
C	−1.172160	−0.430422	−0.046611
C	−2.635310	−0.732885	0.018609
H	−2.917139	−1.315486	0.905639
H	−3.198932	0.195824	0.037469
H	−2.947419	−1.338356	−0.837371
C	−0.678653	0.890859	−0.008811
O	−1.340435	1.933757	0.026559
N	0.723737	1.053131	−0.037981
H	1.087845	1.980022	0.000279
C	1.632099	0.058423	0.010289
O	2.827408	0.215345	0.009639
H	1.845763	−1.935087	0.055849
		CI(S_{CT}/S_0)	
N	1.162334	1.042346	−0.263248
C	0.032843	1.331628	−0.886719
H	0.071177	2.052965	−1.704964
C	−1.168235	0.700011	−0.381541
C	−1.406077	1.232412	1.064511
H	−1.516797	2.315505	1.051847
H	−2.359231	0.817655	1.396950
H	−0.646964	0.980334	1.816615
C	−1.030796	−0.740726	−0.296916
O	−1.865129	−1.605291	−0.182635
N	0.327974	−1.149773	−0.123341
H	0.485579	−2.113117	0.093964
C	1.377938	−0.317455	0.117311
O	2.451642	−0.658032	0.524231
H	2.007931	1.570014	−0.380930
		S_{NP}−Min	
N	1.118267	−1.237341	0.044739
C	−0.253141	−1.518689	0.007675
H	−0.538725	−2.551897	0.056292
C	−1.168598	−0.446918	−0.022008
C	−2.653941	−0.703861	−0.021797
H	−3.013310	−0.910705	0.986002
H	−3.209466	0.143430	−0.420720
H	−2.872031	−1.575108	−0.636353
C	−0.626645	0.797678	−0.036345
O	−1.340757	1.973560	0.013733
N	0.743315	1.035192	0.086639
H	1.104085	1.939825	−0.129636
C	1.656128	0.009531	0.011584
O	2.840365	0.216224	−0.059349

H	1.778689	−1.975222	−0.044953
STC(S_{NP}/T_{NP}/T_{CT})			
N	1.120795	−1.233804	0.048828
C	−0.250053	−1.516763	0.011604
H	−0.532149	−2.551886	0.045354
C	−1.164688	−0.455414	−0.035941
C	−2.652419	−0.702201	−0.016968
H	−3.047691	−0.634323	0.996749
H	−3.181849	0.013410	−0.644895
H	−2.864815	−1.702218	−0.389277
C	−0.625705	0.798493	−0.081720
O	−1.353355	1.961125	0.051529
N	0.742618	1.039028	0.061322
H	1.105331	1.936981	−0.179572
C	1.656466	0.013888	0.012628
O	2.842204	0.219481	−0.042965
H	1.784882	−1.971398	−0.005539
T_{CT}−Min			
N	1.136037	−1.243013	0.055026
C	−0.202653	−1.580820	−0.112823
H	−0.487456	−2.597956	0.108384
C	−1.179229	−0.433155	0.024707
C	−2.637720	−0.717310	0.012641
H	−2.914037	−1.303114	0.893253
H	−3.221890	0.199103	−0.011243
H	−2.882069	−1.323233	−0.862983
C	−0.660316	0.910617	0.002722
O	−1.350881	1.909689	0.001241
N	0.720175	1.040218	−0.015701
H	1.093182	1.966793	−0.017239
C	1.644709	0.020567	0.005134
O	2.826107	0.245090	−0.001860
H	1.828229	−1.959657	0.025211
STC(T_{CT}/S_0)			
N	1.162334	1.042346	−0.263248
C	0.032843	1.331628	−0.886719
H	0.071177	2.052965	−1.704964
C	−1.168235	0.700011	−0.381541
C	−1.406077	1.232412	1.064511
H	−1.516797	2.315505	1.051847
H	−2.359231	0.817655	1.396950
H	−0.646964	0.980334	1.816615
C	−1.030796	−0.740726	−0.296916
O	−1.865129	−1.605291	−0.182635
N	0.327974	−1.149773	−0.123341
H	0.485579	−2.113117	0.093964
C	1.377938	−0.317455	0.117311
O	2.451642	−0.658032	0.524231
H	2.007931	1.570014	−0.380930

6.2. Thymine Oligomer

**S_0–Min**			
N	7.389460	1.855900	3.092680
C	6.702690	2.958980	3.581810
H	6.542360	3.750980	2.867290
C	6.254640	3.047610	4.844870
C	5.515260	4.253660	5.335770
H	4.496130	4.001500	5.609210
H	5.991650	4.669440	6.215880
H	5.486430	5.015550	4.559710
C	6.538180	1.932900	5.758580
O	6.159860	1.883320	6.903930
N	7.283710	0.900060	5.210070
H	7.643540	0.191190	5.841610
C	7.750570	0.818530	3.920750
O	8.401140	−0.127150	3.545370
N	3.784100	−0.237640	2.745370
C	3.504820	1.115640	2.489750
H	3.525040	1.406930	1.451460
C	3.196260	2.017690	3.445230
C	2.850160	3.446920	3.138070
H	1.771080	3.580120	3.118130
H	3.252490	4.129670	3.878740
H	3.254930	3.731830	2.170060
C	3.112730	1.530990	4.825590
O	2.718440	2.163680	5.778470
N	3.541460	0.241170	4.996350
H	3.480570	−0.143250	5.928400
C	3.898300	−0.666660	4.035240
O	4.232390	−1.781710	4.356930
H	7.861800	2.030600	2.220970
H	4.266270	−0.773280	2.020570
CI(S_{CT}/S_{NP})			
N	7.389460	1.855900	3.092680
C	6.702690	2.958980	3.581810
H	6.542360	3.750980	2.867290
C	6.254640	3.047610	4.844870
C	5.515260	4.253660	5.335770
H	4.496130	4.001500	5.609210
H	5.991650	4.669440	6.215880
H	5.486430	5.015550	4.559710
C	6.538180	1.932900	5.758580
O	6.159860	1.883320	6.903930
N	7.283710	0.900060	5.210070
H	7.643540	0.191190	5.841610
C	7.750570	0.818530	3.920750
O	8.401140	−0.127150	3.545370
N	3.784100	−0.237640	2.745370
C	3.504820	1.115640	2.489750
H	3.525040	1.406930	1.451460
C	3.196260	2.017690	3.445230
C	2.850160	3.446920	3.138070

H	1.771080	3.580120	3.118130
H	3.252490	4.129670	3.878740
H	3.254930	3.731830	2.170060
C	3.112730	1.530990	4.825590
O	2.718440	2.163680	5.778470
N	3.541460	0.241170	4.996350
H	3.480570	−0.143250	5.928400
C	3.898300	−0.666660	4.035240
O	4.232390	−1.781710	4.356930
H	7.861800	2.030600	2.220970
H	4.266270	−0.773280	2.020570
	S_{CT}–Min		
N	7.413070	1.916150	3.070240
C	6.619820	2.947210	3.411380
H	6.509630	3.763250	2.722110
C	6.159260	2.994840	4.851390
C	5.482190	4.233430	5.335590
H	4.457380	4.018790	5.628940
H	5.980740	4.642210	6.208600
H	5.463300	4.994720	4.557940
C	6.440900	1.933070	5.705060
O	6.084060	1.809880	6.888890
N	7.206770	0.861770	5.168790
H	7.582470	0.184130	5.822030
C	7.733030	0.832870	3.933820
O	8.428550	−0.066080	3.511120
N	3.840340	−0.226860	2.782140
C	3.585490	1.132720	2.527640
H	3.623100	1.428320	1.491210
C	3.255300	2.028430	3.481820
C	2.898870	3.453390	3.167730
H	1.818640	3.576220	3.138120
H	3.289150	4.134640	3.914180
H	3.310050	3.740510	2.202730
C	3.138080	1.537130	4.858750
O	2.717010	2.169420	5.799880
N	3.541150	0.238620	5.030200
H	3.469020	−0.146640	5.960370
C	3.904350	−0.667930	4.072970
O	4.229160	−1.787220	4.392300
H	7.893930	2.035310	2.182750
H	4.331690	−0.767110	2.068150
	CI(S_{CT}/S_0)		
N	7.461908	1.872182	2.968294
C	6.332719	2.501772	3.282239
H	5.760650	2.955940	2.482847
C	6.125641	2.802804	4.707588
C	7.232793	3.852185	4.991311
H	7.386418	4.486333	4.122919
H	6.842934	4.481270	5.790575
H	8.204107	3.451099	5.300025
C	6.392449	1.700391	5.620707

O	6.110689	1.627259	6.789222
N	7.213685	0.702596	5.041884
H	7.669179	0.067136	5.693455
C	7.880051	0.825860	3.848761
O	8.719365	0.048266	3.488104
N	3.916343	−0.040022	2.810175
C	3.612872	1.318625	2.585392
H	3.614374	1.628444	1.552867
C	3.305007	2.192778	3.569290
C	3.011845	3.645479	3.330565
H	1.957946	3.867557	3.493944
H	3.614266	4.257680	3.999507
H	3.263702	3.919942	2.310036
C	3.238476	1.678148	4.942322
O	2.837146	2.285331	5.904403
N	3.661633	0.377022	5.074098
H	3.649744	−0.021402	6.002484
C	4.039895	−0.493502	4.090098
O	4.404607	−1.611934	4.372987
H	7.968134	2.130651	2.120613
H	4.410931	−0.553438	2.080028
	CI(S_{CT}/S_0)−2		
N	7.092819	1.862964	2.950173
C	5.972327	2.572264	3.268027
H	5.714261	3.348357	2.562011
C	5.633029	2.775116	4.700205
C	5.444863	4.172356	5.241089
H	4.478930	4.286200	5.729809
H	6.198135	4.384011	5.993266
H	5.510364	4.921654	4.456514
C	6.191023	1.798739	5.628049
O	5.950604	1.774513	6.812458
N	7.051366	0.844927	5.075676
H	7.548846	0.248200	5.731607
C	7.682728	0.981455	3.864943
O	8.654120	0.342002	3.569523
N	4.239793	−0.115547	2.756919
C	4.153365	1.314436	2.581210
H	3.891267	1.641389	1.590241
C	3.679873	2.119335	3.677872
C	3.031697	3.435562	3.361782
H	1.972604	3.258297	3.172674
H	3.125399	4.149377	4.170456
H	3.459896	3.874331	2.463299
C	3.331519	1.463112	4.947843
O	2.769398	2.004175	5.870631
N	3.714135	0.152958	5.014836
H	3.568145	−0.324440	5.892008
C	4.193294	−0.649770	3.999507
O	4.464991	−1.800330	4.264672
H	7.617417	2.229002	2.157717
H	4.749831	−0.632841	2.044065

	CPD		
N	7.101791	1.609000	2.847776
C	5.825741	2.230874	3.020127
H	5.786342	3.090371	2.358397
C	5.410612	2.632093	4.433457
C	5.728101	4.046883	4.894672
H	5.139250	4.308104	5.771494
H	6.775694	4.092482	5.177419
H	5.553579	4.783134	4.114382
C	5.996295	1.689093	5.486422
O	5.640901	1.716471	6.636235
N	6.982899	0.822828	5.072048
H	7.471080	0.307731	5.799977
C	7.678453	0.888955	3.867885
O	8.715833	0.301321	3.739653
N	4.320266	0.006196	2.932808
C	4.413505	1.466758	2.792149
H	3.967704	1.757462	1.847310
C	3.889988	2.300493	3.994569
C	3.105922	3.524104	3.532119
H	2.135283	3.221532	3.145122
H	2.956237	4.231172	4.342294
H	3.633958	4.047328	2.736174
C	3.212436	1.551693	5.093935
O	2.562138	2.073835	5.961660
N	3.499478	0.221380	5.117981
H	3.262670	−0.285491	5.958276
C	4.091837	−0.559360	4.143643
O	4.278678	−1.727036	4.400987
H	7.730739	2.114938	2.226094
H	4.829189	−0.541966	2.244655
	S_{NP}–Min		
N	−2.130441	−0.227435	−1.462596
C	−1.284919	−1.333171	−1.271549
H	−1.067406	−1.911489	−2.149799
C	−0.906544	−1.706393	0.025454
C	−0.123397	−2.969276	0.248725
H	0.864902	−2.777190	0.651068
H	−0.639274	−3.614682	0.950078
H	−0.019141	−3.500679	−0.693341
C	−1.341410	−0.904139	1.037464
O	−0.994244	−1.088403	2.356669
N	−2.061471	0.267285	0.814445
H	−2.553709	0.703085	1.586137
C	−2.576355	0.553794	−0.426862
O	−3.328865	1.488152	−0.583888
N	1.181810	2.076716	−0.982903
C	1.588692	0.860328	−1.561082
H	1.611320	0.842560	−2.639281
C	1.968897	−0.221425	−0.851242
C	2.460470	−1.488140	−1.490296
H	3.546948	−1.525303	−1.467845

H	2.082017	−2.366385	−0.980508
H	2.128058	−1.543972	−2.523811
C	1.976208	−0.097666	0.608464
O	2.414592	−0.917169	1.386401
N	1.421309	1.058756	1.081482
H	1.426809	1.195850	2.081500
C	0.994723	2.146114	0.365438
O	0.549585	3.106889	0.947370
H	−2.588778	−0.218846	−2.357520
H	0.663070	2.733883	−1.569168
STC(S_{NP}/T_{NP}/T_{CT})			
N	−2.096359	−0.150056	−1.480014
C	−1.332952	−1.313726	−1.273419
H	−1.103355	−1.891065	−2.148509
C	−0.983793	−1.709662	0.017360
C	−0.205635	−2.978953	0.234887
H	0.781158	−2.784664	0.641252
H	−0.719663	−3.630712	0.931652
H	−0.090541	−3.509176	−0.707511
C	−1.420003	−0.907741	1.037685
O	−1.019023	−1.079945	2.344288
N	−2.060320	0.312725	0.810398
H	−2.612227	0.716347	1.561533
C	−2.547803	0.623205	−0.438081
O	−3.286895	1.568549	−0.591908
N	1.246600	2.063552	−0.965505
C	1.627721	0.842147	−1.549815
H	1.653097	0.830224	−2.627968
C	1.977938	−0.253826	−0.846386
C	2.436606	−1.528376	−1.494676
H	3.521404	−1.603290	−1.467519
H	2.024044	−2.398858	−0.997851
H	2.108671	−1.561411	−2.530404
C	1.975137	−0.142554	0.614441
O	2.384819	−0.980852	1.387903
N	1.444950	1.023445	1.092315
H	1.454349	1.153541	2.093206
C	1.049119	2.126460	0.381846
O	0.622223	3.092618	0.967829
H	−2.576410	−0.150893	−2.364980
H	0.748368	2.737481	−1.551192
T_{CT}–Min			
N	−2.027972	0.241743	−1.595983
C	−1.256895	−0.911459	−1.448986
H	−1.184390	−1.538932	−2.325052
C	−1.285211	−1.565958	−0.089302
C	−0.657329	−2.908122	0.057017
H	0.325400	−2.827524	0.516717
H	−1.249808	−3.561312	0.686340
H	−0.535299	−3.368034	−0.921158
C	−1.762475	−0.800084	1.036366
O	−1.653249	−1.164434	2.189203

N	−2.339634	0.430450	0.731157
H	−2.846735	0.913674	1.468304
C	−2.555005	0.939115	−0.530549
O	−3.147930	1.982116	−0.673783
N	1.781503	1.947406	−0.788880
C	2.027821	0.712291	−1.416311
H	2.175462	0.753012	−2.484043
C	2.104498	−0.462647	−0.756612
C	2.398387	−1.772004	−1.432412
H	3.446731	−2.038825	−1.317020
H	1.798053	−2.573375	−1.014551
H	2.164803	−1.710994	−2.492185
C	1.971587	−0.423780	0.702639
O	2.161065	−1.355713	1.452189
N	1.581933	0.787784	1.205033
H	1.507395	0.865401	2.209484
C	1.447736	1.974355	0.532298
O	1.108504	2.966470	1.131256
H	−2.375270	0.393199	−2.530623
H	1.457043	2.724277	−1.371306
	STC(T_{CT}/S_0)		
N	7.121285	1.611163	2.938393
C	5.949321	2.361571	3.277970
H	5.900410	3.250085	2.659563
C	5.897490	2.702939	4.724084
C	5.375656	4.005024	5.244361
H	4.353299	3.920197	5.604904
H	5.976232	4.325151	6.089003
H	5.404632	4.771684	4.473778
C	6.417781	1.719850	5.674316
O	6.199819	1.774420	6.860774
N	7.172164	0.700533	5.113463
H	7.695895	0.114683	5.761203
C	7.723322	0.764204	3.845672
O	8.660805	0.066843	3.554429
N	4.267988	0.070416	2.894243
C	4.333315	1.520728	2.738070
H	4.241159	1.785117	1.692936
C	3.318585	2.221091	3.557615
C	2.822930	3.577308	3.172655
H	1.741001	3.560527	3.062325
H	3.072729	4.335145	3.911333
H	3.261014	3.883956	2.225197
C	3.060504	1.666854	4.878536
O	2.464882	2.227217	5.772846
N	3.599553	0.421656	5.087423
H	3.400226	−0.019495	5.973362
C	4.117155	−0.438897	4.142226
O	4.331147	−1.586624	4.451211
H	7.688733	2.050390	2.224154
H	4.778288	−0.488936	2.215524

References

1. Mees, A.; Klar, T.; Gnau, P.; Hennecke, U.; Eker, A.P.M.; Carell, T.; Essen, L.O. Crystal structure of a photolyase bound to a CPD-like DNA lesion after in situ repair. *Science* **2004**, *306*, 1789–1793.
2. Case, D.A.; Darden, T.A.; Cheatham, T.E., III; Simmerling, C.L.; Wang, J.; Duke, R.E.; Luo, R.; Merz, K.M.; Pearlman, D.A.; Crowley, M.; et al. *AMBER 10*; University of California: San Francisco, CA, USA, 2008.
3. Wang, J.M.; Cieplak, P.; Kollman, P.A. How well does a restrained electrostatic potential (RESP) model perform in calculating conformational energies of organic and biological molecules? *J. Comput. Chem.* **2000**, *21*, 1049–1074.
4. Ponder, J.W.; Richards, F.M. An efficient newton-like method for molecular mechanics energy minimization of large molecules. *J. Comput. Chem*. **1987**, *8*, 1016–1024.
5. Ferré, N.; Cembran, A.; Garavelli, M.; Olivucci, M. Complete-active-space self-consistent-field/Amber parameterization of the Lys296-retinal-Glu113 rhodopsin chromophore-counterion system. *Theor. Chem. Acc.* **2004**, *112*, 335–341.
6. Ferré, N.; Olivucci, M. Probing the rhodopsin cavity with reduced retinal models at the CASPT2//CASSCF/AMBER Level of theory. *J. Am. Chem. Soc.* **2003**, *125*, 6868–6869.
7. Andruniow, T.; Ferré, N.; Olivucci, M. Structure, initial excited-state relaxation, and energy storage of rhodopsin resolved at the multiconfigurational perturbation theory level. *Proc. Natl. Acad. Sci. USA* **2004**, *101*, 17908–17913.
8. Luo, G.F.; Chen, X.B. Ground-state intermolecular proton transfer of N_2O_4 and H_2O: An important source of atmospheric hydroxyl radical? *J. Phys. Chem. Lett.* **2012**, *3*, 1147–1153.
9. Roos, B.O.; Taylor, P.R.; Siegbahn, P.E.M. A complete active space SCF method (CASSCF) using a density matrix formulated super-CI approach. *Chem. Phys*. **1980**, *48*, 157–173.
10. Ruedenberg, K.; Schmidt, M.W.; Gilbert, M.M.; Elbert, S.T. Are atoms intrinsic to molecular electronic wavefunctions? I. The FORS model. *Chem. Phys*. **1982**, *71*, 41–49.
11. Andersson, K.; Malmqvist, P.Å.; Roos, B.O.; Sadlej, A.J.; Wolinski, K. Second-order perturbation theory with a CASSCF reference function. *J. Phys. Chem*. **1990**, *94*, 5483–5488.
12. Andersson, K.; Malmqvist, P.Å.; Roos, B.O. Second-order perturbation theory with a complete active space self-consistent field reference function. *J. Chem. Phys*. **1992**, *96*, 1218–1226.
13. Fukui, K. The path of chemical reactions—The IRC approach. *Acc. Chem. Res*. **1981**, *14*, 363–368.
14. Hratchian, H.P.; Schlegel, H.B. Accurate reaction paths using a Hessian based predictor-corrector integrator. *J. Chem. Phys*. **2004**, *12*, 9918–9924.
15. Frisch, M.J.; Trucks, G.W.; Schlegel, H.B.; Scuseria, G.E.; Robb, M.A.; Cheeseman, J.R.; Montgomery, J.A., Jr.; Vreven, T.; Kudin, K.N.; Burant, J.C.; *Gaussian03*, revision D.02; Gaussian, Inc.: Pittsburgh, PA, USA, 2004.
16. Aquilante, F.; De Vico, L.; Ferré, N.; Ghigo, G.; Malmqvist, P.-Å.; Neogrády, P.; Pedersen, T.B.; Pitoňák, M.; Reiher, M.; Roos, B.O.; et al. MOLCAS 7: The Next Generation. *J. Comput. Chem*. **2010**, *31*, 224.
17. Ferré, N.; Cembran, A.; Garavelli, M.; Olivucci. M. Complete-active-space self-consistent-field/Amber parameterization of the Lys296–retinal–Glu113 rhodopsin chromophore-counterion system. *Theor. Chem. Acc*. **2004**, *112*, 335–341.
